# Diversity of Culturable Fungi in Two-Phase Olive Mill Waste, a Preliminary Evaluation of Their Enzymatic Potential, and Two New *Trichoderma* Species

**DOI:** 10.3390/jof11090687

**Published:** 2025-09-22

**Authors:** Vassiliki Fryssouli, Io Kefalogianni, Elias Polemis, Milton A. Typas, Georgios I. Zervakis

**Affiliations:** 1 Laboratory of General and Agricultural Microbiology, Agricultural University of Athens, Iera Odos 75, 11855 Athens, Greece; vfrisouli@gmail.com (V.F.); kefalogianniio@gmail.com (I.K.); eliasp@ath.forthnet.gr (E.P.); 2 Section of Genetics and Biotechnology, Department of Biology, National and Kapodistrian University of Athens, Panepistemiopolis, 15701 Athens, Greece; matypas@biol.uoa.gr

**Keywords:** Ascomycota, biodegradation of lignocellulosics, effluent detoxification, fungal diversity, new species, olive mill waste, phylogeny, *Trichoderma*, wastes valorization

## Abstract

This study investigates the diversity and provides a preliminary evaluation of the enzymatic potential of culturable fungi present in two-phase olive mill waste (TPOMW), a lignocellulose- and phenolic-rich agro-industrial by-product generated in large quantities in olive oil-producing countries. Ninety-four isolates, representing 31 species of the phyla Ascomycota, Basidiomycota, and Mucoromycota, were obtained and identified by using ITS, 28S, *tef*1-α, *tub*2, *rpb*2, *act*, and/or *cal* sequences. Among the identified taxa, two new *Trichoderma* species within the Harzianum clade, namely *Trichoderma amurcicola* (phylogenetically related to *T. simile* and *T. guizhouense*) and *Trichoderma olivarum* (phylogenetically related to *T. simmonsii*), were described following a multilocus phylogenetic analysis combined with a study of their morphoanatomical features. A rather high phylogenetic divergence was detected in *Candida boidinii*, *Pleurostoma richardsiae,* and *Mucor circinelloides*, while *Cladosporium limoniforme*, *Mucor pseudolusitanicus*, *Stagonosporopsis ailanthicola,* and *Talaromyces nanjingensis* were recorded for the first time in TPOMW. A preliminary screening revealed 29 species with cellulolytic and/or xylanolytic activities; 26 species displayed dye decolorization capacity, while ligninolytic and laccase activities were restricted to a few taxa. The most promising degraders of lignocellulosics included strains of *Cladosporium limoniforme*, *C. ramotenellum*, *Fuscoporia ferrea, Peniophora lycii*, and *Pseudophlebia setulosa*. Fungi detected in TPOMW are promising biotechnological tools to be exploited in the frame of circular economy applications.

## 1. Introduction

Olive cultivation and olive oil are cornerstones of Mediterranean agriculture, economy, and nutrition. However, the olive oil extraction process is linked with the generation of huge quantities of olive mill wastewater, a by-product with significant environmental repercussions due to its high organic load and chemical composition [1,2]. Disposal of improperly managed olive mill wastewater can lead to contamination of soil and water receptors, disruption of ecosystem functions, and public health risks [3,4]. Consequently, sustainable waste management strategies have become a priority, aiming to align with circular economy principles while mitigating both ecological and socio-economic impacts [5,6].

**Figure 1:**
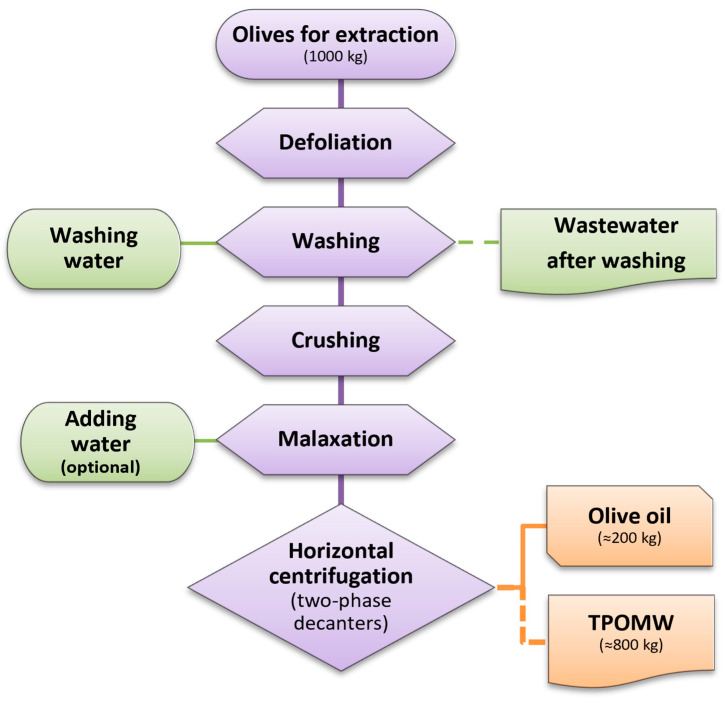
Schematic representation of the two-phase olive oil extraction process and the generation of two-phase olive mill waste (TPOMW). Waste streams, including wastewater from olive washing and TPOMW, are depicted by dashed lines.

Towards this end, the relatively recent transition leading to the wide use of two-phase olive oil extraction technology has significantly reduced the volume of waste generated; the latter is a sludge-like effluent widely known as ‘alpeorujo’ or two-phase olive mill waste (TPOMW) (Figure 1). It is rich in polyphenols, fatty acids, and proteins [7,8], while it is also characterized by high chemical oxygen demand (COD) and biological oxygen demand (BOD) values, which render it as a pollutant with severe adverse environmental impact. Furthermore, TPOMW’s seasonal production and composition heterogeneity further complicate its effective management. Therefore, innovative strategies are essential for mitigating these risks while unlocking the exploitation potential of this waste stream.

In general, olive mill waste (OMW) presents significant opportunities for sustainable applications [9]; recent advances in biotechnology have enabled the recovery of bioactive compounds for use in agriculture (e.g., biostimulants, biopesticides), and in the food and pharmaceutical industries. In particular, the lignocellulosic fraction of TPOMW could be transformed through the use of microbial extracellular enzymes (cellulases, hemicellulases, lignin-modifying enzymes) into fermentable sugars, biofuels, and value-added molecules [10,11]. For example, fungal taxa within Ascomycota (e.g., *Trichoderma*, *Aspergillus*), Basidiomycota (e.g., white-rot species), and Mucoromycota (e.g., *Mucor*, *Rhizopus*) secrete a broad spectrum of hydrolytic and oxidative enzymes that target both polysaccharidic and aromatic fractions of OMW [12,13]. Concurrently, bacterial phyla—such as Firmicutes (e.g., *Bacillus* spp.), Proteobacteria (e.g., *Pseudomonas*, *Acinetobacter*), and Actinobacteria (e.g., *Streptomyces*, *Arthrobacter*)—contribute complementary hydrolytic and oxidative capabilities, particularly in anaerobic or microaerophilic microenvironments [1,14]. Furthermore, microbial communities, which are adapted to the toxic and recalcitrant nature of OMW, exhibit enhanced efficiency in degrading complex organic substrates [15,16,17]. However, the diversity and enzymatic potential of these communities remain largely underexplored [4,18,19], particularly of those existing in TPOMW [20,21,22], thus leaving significant opportunities untapped for developing its sustainable valorization. This approach aligns with circular economy principles, transforming waste into high-value products while addressing environmental concerns [23].

Fungal communities present in OMW have been extensively investigated over the past two decades, with a gradual methodological shift from classical morphology-based identification to molecular and metagenomic approaches. Early culture-based surveys identified yeast species, including *Candida boidinii* and *Geotrichum candidum,* in untreated TPOMW [20]. Subsequent studies reported filamentous taxa, such as *Penicillium roqueforti*, *Cladosporium* spp., and yeasts of the genera *Pichia* and *Candida,* in both raw and composted TPOMW [19,21]. Similar taxonomic profiles were observed in OMW and other related substrates, where members of Ascomycota, mainly Eurotiales and Hypocreales, prevail, whereas Basidiomycota are infrequently reported and occurrences of Chytridiomycota (e.g., *Rhizophydium* spp.) remain rare and sporadic [1,4,15,17,19,20,21,22,24].

This study aims to contribute to the knowledge of the diversity of culturable fungi present in TPOMW. A large variety of members from this particular fungal community were obtained using diverse selective solid media, inoculation techniques, and culture conditions. In addition, by integrating molecular and biochemical approaches, we aimed (a) to elucidate the taxonomic diversity of fungi existing in TPOMW through multilocus phylogenetic analyses and (b) to perform a preliminary/initial evaluation of the strains’ lignocellulolytic and dye-decolorization activities aiming at their future exploitation.

## 2. Materials and Methods

### 2.1. Fungal Isolation from TPOMW, Selection, and Cultivation Conditions

TPOMW samples were collected from an olive mill equipped with two-phase centrifugal decanters in Kalamata, Greece, during three production seasons. Following samples homogenization, 10 g from each were suspended in 100 mL sterile saline solution (0.9% NaCl), blended for 2 min, and passed through sterile gauze to remove coarse solids. The resulting homogenate was used for the screening and selection of culturable fungi on the following media: (a) carboxymethyl cellulose-enriched agar (CEA), (b) lignin-enriched agar (LEA), (c) yeast peptone dextrose agar (YPDA), and (d) potato dextrose agar (PDA, Condalab, Madrid, Spain) (Supplementary Materials, Table S1). All media were sterilized at 121 °C for 20 min. To suppress fast-growing mitosporic fungi, the ergosterol synthesis inhibitor econazole nitrate (Spectazole^®^, Janssen Pharmaceuticals, Beerse, Belgium) was added to the above media at concentrations of 10 and 25 mg L^−1^. Additionally, PDA supplemented with 0.04% (*v*/*v*) Remazol Brilliant Blue R (RBBR; Sigma-Aldrich, St. Louis, MO, USA) was used to enrich phenol-degrading fungi and to limit bacterial growth.

Three complementary isolation strategies were adopted: (a) serial dilutions: ten-fold serial dilutions (10^−1^ to 10^−6^) were prepared from the homogenate; (b) pre-enrichment cultures: 1 g of TPOMW was incubated in 50 mL of LEM (lignin-enriched medium), CEM (cellulose-enriched medium), or YPDB (yeast peptone dextrose broth) at 25 °C, 130 rpm for 48 h, and the resulting biomass was diluted and plated as above; (c) modified Warcup plate method: approximately 1.5 g of TPOMW was evenly distributed in 90 mm sterile Petri dishes and overlaid with 20 mL of liquefied, cooled agar medium (CEA, LEA, or YPDA; with or without econazole), followed by gentle swirling to ensure contact with the underlying substrate.

In all cases, aliquots of 100 μL from 10^−3^ to 10^−5^ suspensions were spread on agar plates (prepared in triplicate) to ensure that single colonies could develop for subsequent isolation. All plates were incubated in the dark under mesophilic (25 °C) and thermophilic (45 °C) temperature regimes for 14 days. The latter temperature was selected for isolating thermophilic fungi possibly present in TPOMW; it should be noted that during the extraction process, it is common practice to add hot water during milling and malaxation of olives to increase oil extraction efficiency. Emerging colonies were transferred for subculturing in PDA and grouped into morphological operational taxonomic units (MOTUs) based on their macro- and micromorphological characteristics, including colony morphology (e.g., color, growth rate, pigment production), microscopic features (e.g., hyphal morphology, spore-bearing structures), and isolation conditions for fungi lacking distinct morphological features (e.g., yeasts).

A total of 56 distinct MOTUs were retained, and representative isolates from each one were selected for molecular identification and biodegradation assays; pertinent information, including the marker(s) used to identify species for each genus represented by the MOTUs, are detailed in Table 1. All isolates were preserved in 30% (*v*/*v*) glycerol prepared with 0.85% (*w*/*v*) NaCl, and stored at −80 °C.

### 2.2. DNA Extraction, PCR, and Sequencing

Genomic DNA was extracted from colonies grown on PDA (3–5 days at 25 °C) for filamentous fungi and in YPDB (1–2 days at 30 °C and 120 rpm) for yeasts using a cetyltrimethylammonium bromide (CTAB)-based protocol provided by the NucleoSpin^®^ Plant II kit (Macherey-Nagel GmbH, Düren, Germany). DNA quality and concentration were assessed spectrophotometrically (NanoDrop ND-1000, Thermo Fisher Scientific, Waltham, MA, USA), and integrity was confirmed by 1% agarose gel electrophoresis.

Seven nuclear loci were targeted for amplification: the internal transcribed spacer region (ITS), the large subunit ribosomal *RNA* gene (28S; domains D1 and D2), and partial fragments of translation elongation factor 1-α (*tef*1-α), β-tubulin (*tub*2), RNA polymerase II second largest subunit (*rpb*2), actin (*act*), and calmodulin (*cal*). ITS served as the primary molecular marker for the identification of fungi, while additional markers were employed when ITS was not sufficient alone to provide the necessary phylogenetic resolution and taxonomic accuracy for determining the identity of strains under study. The selection of additional markers was based on pertinent literature and on the availability of the respective reference sequences in public databases. Details on the markers used for each fungal genus are provided in Table 1.

PCRs were performed in a 25 µL reaction volume containing 1 × PCR buffer, 2.5 mM MgCl_2_, 0.25 mM of each dNTP, 0.25 µM of each primer, 1 U Platinum^®^ Taq DNA Polymerase (Invitrogen, Carlsbad, CA, USA), and 10–50 ng of genomic DNA. Thermal cycling was performed in a MiniAmp Plus Thermal Cycler (Applied Biosystems, Foster City, CA, USA) under the following conditions: initial denaturation at 94 °C for 3 min; 35 cycles of 94 °C for 30 s, annealing temperature (as described in Supplementary Materials, Table S2) for 30 s, and 72 °C for 1 min; and final extension at 72 °C for 7 min. Amplification products were visualized on 1% agarose gels stained with ethidium bromide (EtBr; Sigma-Aldrich, St. Louis, MO, USA) and photographed under UV illumination. The primer sequences, expected amplicon sizes, and pertinent references are also listed in Table S2.

Amplicons of expected sizes were excised and purified using the PureLink^®^ PCR Purification Kit (Thermo Fisher Scientific, Waltham, MA, USA). Sanger sequencing was performed in both directions by CEMIA Genomics Services (Larissa, Greece) using the same primer pairs as those used for amplification. Chromatograms were inspected and edited manually in Chromas Lite v2.6 (Technelysium Pty Ltd., Tewantin, Australia), and bidirectional reads were assembled into consensus sequences using MEGA v11 [25]. All assembled sequences were queried against the NCBI GenBank database using BLASTn (https://blast.ncbi.nlm.nih.gov/Blast.cgi, accessed on 10 March 2025) to obtain preliminary identifications. Verified sequences were deposited in GenBank, and accession numbers are listed in Table 1.

### 2.3. Phylogenetic Analyses

Phylogenetic analyses were conducted to resolve the taxonomic identity of the fungal strains. Nine datasets were assembled comprising both single-locus (ITS, 28S, *tef*1-α, *tub*2, *rpb*2, *act*, *cal*) and/or multilocus alignments (Supplementary Materials, Table S3). Multiple sequence alignments were generated using MAFFT v7.3 [26], https://mafft.cbrc.jp/alignment/server/, accessed on 25 March 2025, under default settings and manually adjusted in MEGA v11 to minimize gaps and trim the end of the markers. Both maximum likelihood (ML) and Bayesian inference (BI) approaches were employed to reconstruct phylogenetic relationships for each dataset. ML analyses were performed using IQ-TREE v2.2.7 [27] via the CIPRES Science Gateway [28], https://www.phylo.org, accessed on 2 April 2025. Node support was assessed using ultrafast bootstrap approximation (UFBoot) with 10,000 replicates. BI analyses were conducted in MrBayes v3.2.7a [29]. The best-fit models of nucleotide substitution were estimated using jModelTest2 v2.1.6 [30] under the Bayesian information criterion (BIC). Two independent runs of four Markov Chain Monte Carlo (MCMC) chains were performed, with sampling every 1000 generations until the average standard deviation of split frequencies dropped below 0.01. The first 25% of the trees were discarded as burn-in, and a 50% majority-rule consensus tree was constructed.

Phylogenetic trees were visualized and annotated using iTOL v5 [31]. The ML topology is presented in the corresponding figures, with both maximum likelihood bootstrap (MLBS ≥ 65%) and Bayesian posterior probabilities (BPP ≥ 0.95) values displayed at the nodes. All sequence alignments and resulting phylograms were deposited in TreeBASE (www.treebase.org, accessed on 3 July 2025) under accession number 32208.

### 2.4. Morphological Characterization of Isolated Fungi

Microscopic structures were examined on semi-permanent mounts prepared in 3% KOH. Observations were carried out using an Olympus BX53F2 (Olympus Corporation, Tokyo, Japan) or a Zeiss AxioImager A2 (Carl Zeiss Microscopy, Oberkochen, Germany) microscope and were photographed with an Olympus DP74 camera using the cellSens Entry software (Olympus Life Science, Waltham, MA, USA) and with an Axiocam 305 color camera using the ZEN v2.3 lite software (Carl Zeiss Microscopy, Oberkochen, Germany). Photographs were taken under bright field, phase contrast, and/or differential interference contrast (DIC) microscopy.

For the new species of the genus *Trichoderma*, quantitative morphological traits included measurements of phialides (length, width, basal width), supporting cells (width), conidia (length, width), and chlamydospores (length and width, when present). Approximately 70, 50, and 10 measurements from three different biological replicates (cultures per strain) were obtained from phialides, conidia, and chlamydospores, respectively. Τhe following ratios were calculated: (1) l/w = length-to-width ratio for each spore type and phialides; (2) phialide length-to-supporting hyphal width; (3) phialide width-to-supporting hyphal width. Images were edited, and measurements (*n*) were recorded using Fiji v2.7.03 [32]. The results are presented as means and ranges corresponding to 95% of measured values, and the observed minimum and maximum values are placed in parentheses.

Colony growth characteristics were evaluated on two nutrient media: PDA and synthetic nutrient-poor agar (SNA) (Table S1). Agar plugs (5 mm diameter) were excised from actively growing 3-day-old PDA cultures and placed 0.5 cm from the edge of 90 mm Petri dishes. Cultures were incubated at 25 °C, 30 °C, and 35 °C in the dark, and colony diameters were recorded daily until full colonization of the substrate. Each treatment was performed in triplicate.

The microscopic features assessed included colony appearance, radial growth, texture, color, aerial mycelium development, conidial pustule formation, and production of diffusible pigments and/or odor. Stereomicroscopic observations were conducted with a Nikon SMZ18 stereoscope (Nikon Corporation, Tokyo, Japan) with OCULAR v2.0 software (Teledyne Photometrics, Tucson, AZ, USA).

### 2.5. Enzyme Activity and Biodegradation Potential

A subset of 66 representative fungal isolates was screened for enzymatic activity on solid media enriched with various lignocellulosic or chromogenic substrates. The tested parameters included (1) cellulase, xylanase, and ligninase activity on CEA (cellulose), XEA (xylan), and LEA (lignin) media, respectively; (2) laccase activity on PDA supplemented with 0.04% guaiacol (PDA-G); and (3) dye decolorization ability on minimal salt agar (MSA) supplemented with 0.04% Remazol Brilliant Blue R (MS-RB). All media compositions are detailed in Table S1. Assays were conducted in 90 mm Petri dishes incubated at 25 °C in the dark, by means of 5 mm mycelial plugs placed near the edge of the Petri dish. Each test was performed in triplicate.

The enzyme index (EI) was calculated as a proxy for hydrolytic, oxidative, or dye-decolorizing capacity.

For filamentous fungi,

(1) EI_m_ = R_h_/R_c_.

For yeasts,

(2) EI_m_ = R_h_/T,

where Rh is the radius (mm) of the halo zone, Rc the colony radius (mm), and T the number of incubation days [33,34].

Cellulase activity was revealed by halo formation after staining CEA plates with Lugol’s iodine solution (5% iodine) for 3–5 min. Xylan and lignin degradation were inferred from the clear halos formed around fungal colonies developing on the XEA and LEA media, respectively. RBBR decolorization was detected by loss of blue coloration around the colonies. Laccase activity on PDA-G was visualized as reddish-brown pigmentation around the growing mycelium, indicating guaiacol oxidation.

## 3. Results

### 3.1. Diversity and Phylogeny of Fungi from TPOMW

**Figure 2:**
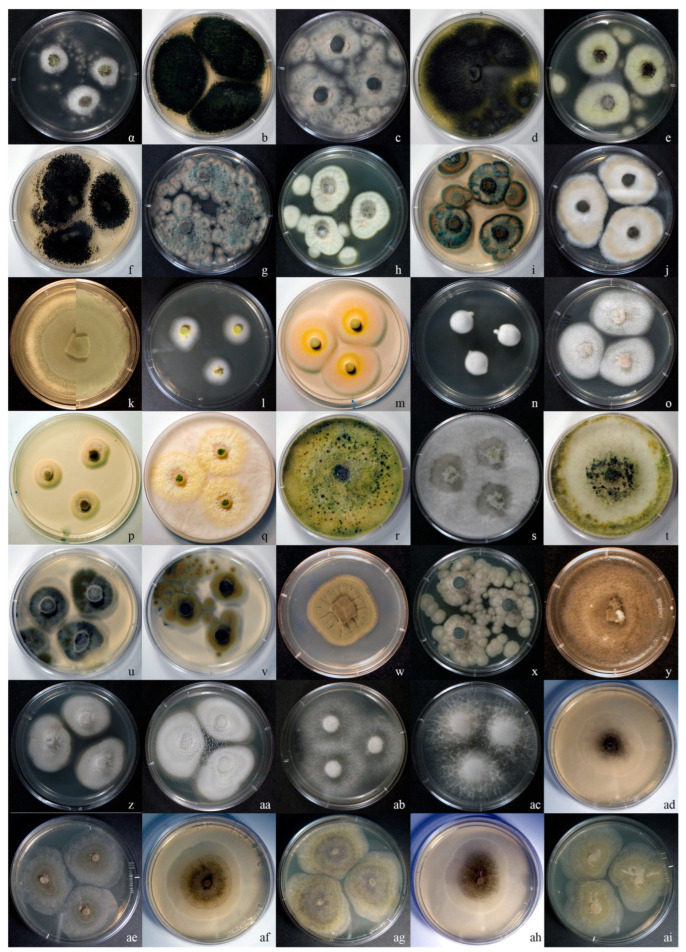
Representative fungal colonies (growing on PDA at 25 °C for 2 to 5 days) derived from isolates obtained from TPOMW. Strain codes are given in parentheses by omitting the common-to-all prefix “LGAM SOW_”. (**a**,**b**) *Aspergillus novoparasiticus* (T2, T1); (**c**,**d**) *A. fumigatus* (A3); (**e**,**f**) *A. welwitschiae* (A4, A8); (**g**) *A. tubingensis* (A6); (**h**) *Penicillium crustosum* (P3); (**i**) *P. kongii* (PT5); (**j**) *P. paneum* (M15); (**k**) *P. roqueforti* (composition of cultures OM122 and OM132); (**l**,**m**) *Talaromyces nanjingensis* (M13); (**n**) *Beauveria pseudobassiana* (BF1); (**o**) *Sarocladium kiliense* (M10); (**p**) *Pleurostoma richardsiae* (M3); (**q**,**r**) *Trichoderma olivarum* (MF1a); (**s**,**t**) *T. amurcicola* (MF2a); (**u**) *Cladosporium cladosporioides* (M4); (**v**) *C. ramotenellum* (PT4); (**w**) *C. limoniforme* (OM52); (**x**) *Neocucurbitaria keratinophila* (M4a); (**y**) *Stagonosporopsis ailanthicola* (OM34); (**z**,**aa**) *Geotrichum candidum* (OM5, GZ3); (**ab**) *Pseudophlebia setulosa* (PT3N); (**ac**) *Fuscoporia ferrea* (M9a); (**ad**,**ae**) *Mucor racemosus* (Z1); (**af**,**ag**) *M. circinelloides* (Z3); (**ah**,**ai**) *M. pseudolusitanicus* (Z7).

**Figure 3:**
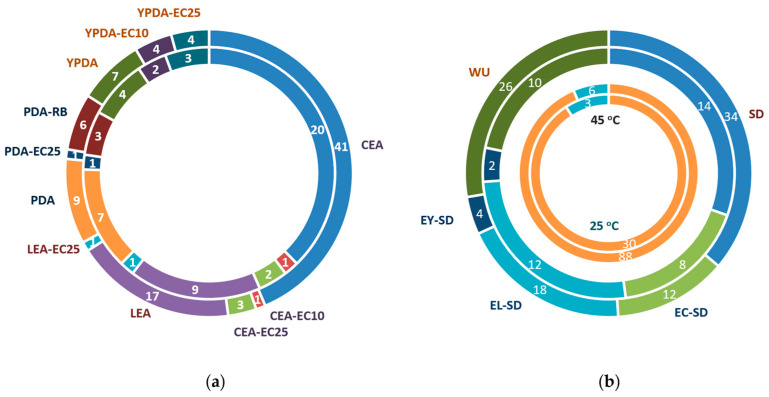
Isolation conditions of the culturable fungal diversity in TPOMW: (**a**) origin of isolates across various substrates (CEA, XEA, LEA, PDA-RB, YPDA), including selective factors (RB: RBBR; EC10/EC25; Table 1). (**b**) Origin of isolates based on inoculation techniques (SD: serial dilution; EC-SD, EL-SD, EY-SD: pre-enrichment in CEA, LEA, YPDB; WU: Warcup method), and incubation temperatures (25 °C, 45 °C). The outer circle represents the number of isolates, while the inner circle indicates the number of identified species.

By using a combination of various substrates, inoculation strategies, and incubation regimes, 94 fungal strains were isolated from TPOMW and grouped into 56 MOTUs (indicative colony morphologies appear in Figure 2). The majority of isolates were recovered from cellulose-enriched media (CEA; 44%) and via serial dilution methods (36%) (Table 2 and Figure 3). Lignin-enriched media (LEA) yielded 19% of the strains, predominantly through the modified Warcup method (72% of them); species like *Aspergillus fumigatus, Penicillium paneum, Fuscoporia ferrea,* and *Peniophora lycii* were isolated only through this method. Incubation under mesophilic conditions (25 °C) led to the isolation of a considerably higher number of strains than under thermophilic conditions (45 °C) (94% vs. 6%, respectively). Among dominant genera, *Aspergillus filifer*, *A. sydowii*, *A. westerdijkiae*, *Cladosporium cladosporioides,* and *C. limoniforme* were each represented by one isolate. In contrast, *Penicillium roqueforti* and *Candida boidinii* were isolated multiple times, possibly reflecting overestimation of MOTU richness and/or their broader ecological amplitude due to their morphological variability and sampling through different isolation treatments.

**Figure 4:**
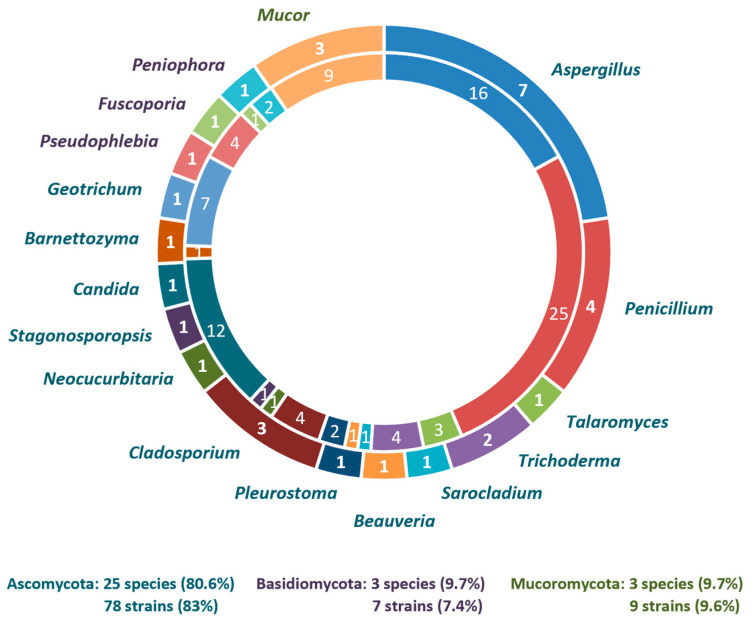
Composition of culturable fungal diversity in TPOMW: number of identified species per fungal genus (outer circle), an number of isolates per genus (inner circle). Distribution of fungal species per phylum is presented by different colors: Ascomycota (blue), Basidiomycota (purple), and Mucoromycota (green).

**Figure 5:**
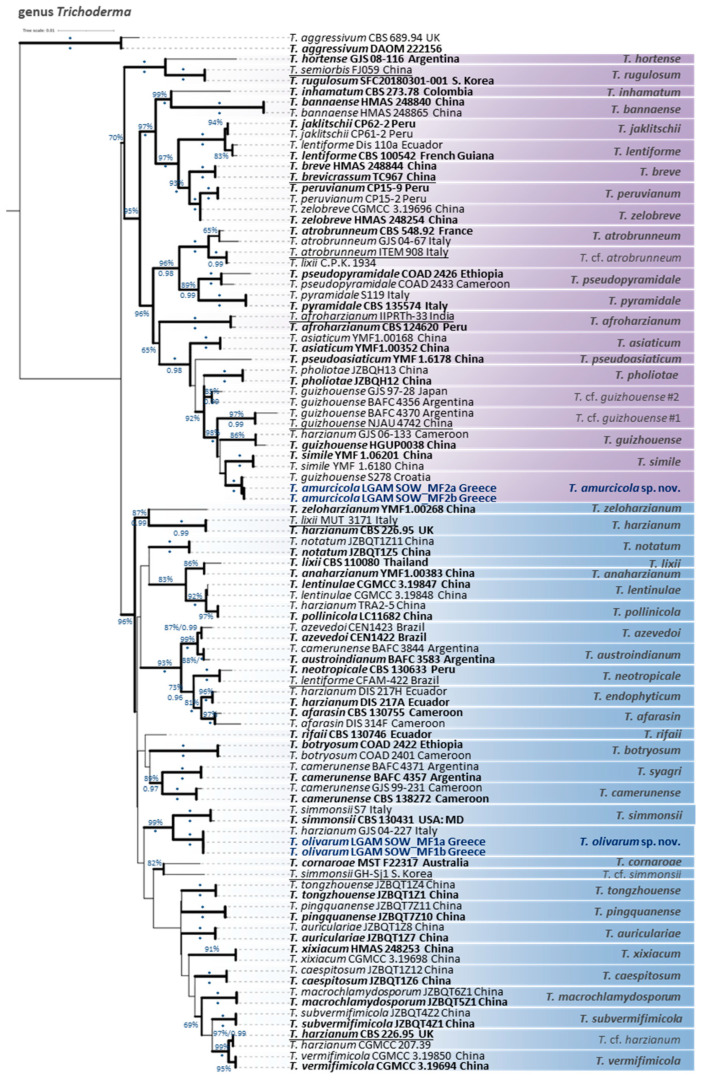
Multilocus phylogenetic tree (ITS, *rpb*2, *tef*1-α, *cal*, *act*) showing the placement of *Trichoderma amurcicola* sp. nov. and *T. olivarum* sp. nov. within the Harzianum clade (in bold blue). Type strains are depicted in bold black, and genome-sequenced strains are underlined. Branch support values are shown, where MLBS ≥ 65% and BPP ≥ 0.95; asterisks (*) denote MLBS = 100% and BPP = 1.00.

Molecular identification of MOTUs was carried out by using one to five markers (out of a total number of seven markers examined), depending on the fungal group under examination; hence, a total of 93 ITS, 1 28S, 22 *tef*1-α, 18 *tub*2, 7 *act*, 6 *rpb*2, and 4 *cal* sequences were generated for the first time (Table 1). In addition, nine datasets were analyzed for several fungal groups in order to resolve taxonomic uncertainties through single or multilocus phylogenetic inference using suitable reference sequences (Figure 4 and Figure 5; Supplementary Materials, Figures S1 and S2, Tables S3–S9). Subsequently, a total of 31 species assigned to 17 genera and 11 orders were identified.

The phylum Ascomycota dominated the culturable mycobiota, representing 83% of the strains isolated, followed by Basidiomycota and Mucoromycota, each accounting for 7% and 10% of the strains, respectively. Ascomycota were represented by 25 species of 13 genera distributed across eight orders: Calosphaeriales, Cladosporiales, Dipodascales, Eurotiales, Hypocreales, Phaffomycetales, Pichiales, and Pleosporales. In Basidiomycota, three species of different genera (i.e., *Fuscoporia*, *Peniophora,* and *Pseudophlebia*) were identified representing the orders Hymenochaetales and Polyporales. All Mucoromycota isolates were grouped into the genus *Mucor* (Mucorales).

More specifically, three species of *Mucor* were recovered: *M. racemosus*, *M. circinelloides*, and *M. pseudolusitanicus* (related to *M. circinelloides*) (Supplementary Materials, Figure S1). The *M. circinelloides* strain was grouped together with several publicly available strains submitted under this name [35], yet it was placed separately from the neotype of the species (CBS 195.68), sharing 99.0% ITS sequence identity. The distinct clustering may reflect ecological adaptation or taxonomic divergence, warranting future research on this taxonomic group. In contrast, strains corresponding to *M. racemosus* and *M. pseudolusitanicus* exhibited >99.8% ITS sequence identity to type-derived sequences and formed well-supported clades.

Three species of Basidiomycota were identified using ITS sequences: *Peniophora lycii* (Peniophoraceae), *Pseudophlebia setulosa* (Meruliaceae), and *Fuscoporia ferrea* (Hymenochaetaceae). Although sequences from type specimens were not available, the isolated strains matched with high identity (>99.5%) to numerous published sequences, e.g., CBS:264.56 [36], AH31879 [37], and CBS:460.86 [36], respectively.

As regards Ascomycota, the family Aspergillaceae (Eurotiales) dominated the culturable fungal diversity in TPOMW, accounting for nearly half of all isolates (44%) and comprising 11 species from the genera *Aspergillus* and *Penicillium*. Species-level identification was achieved primarily through the use of ITS and *tub*2, while *tef*1-α was additionally employed to improve species delimitation in certain cases.

The genus Aspergillus was represented by seven species across five sections: Circumdati (*A. westerdijkiae*), Flavi (*A. novoparasiticus*), Fumigati (*A. fumigatus*), Nidulantes (*A. sydowii*, *A. filifer*), and Nigri (*A. tubingensis*, *A. welwitschiae*) (Supplementary Materials, Figure S2a). Species delimitation within sections Nigri and Flavi was challenging due to high sequence identity among closely related taxa (e.g., *A. welwitschiae* vs. *A. niger*: ITS 99.5%, *tub*2 99.6%; *A. welwitschiae* vs. *A. foetidus*: ITS 100%, *tub*2 99.5%; *A. tubingensis* vs. *A. neoniger*: ITS 100%, *tub*2 99.6%; *A. novoparasiticus* vs. *A. toxicarius*: ITS 99.0%, *tub*2 100%). In contrast, taxa in sections Nudilantes, Fumigati, and Circumdati were rather clearly resolved, e.g., *A. filifer* (including its synonym *A. chinensis*) vs. *A. stellatus* (ITS 100%, *tub*2 93.0%), *A. sydowii* vs. *A. griseoaurantiacus* (ITS 98.4%, *tub*2 96.1%), *A. fumigatus* (including var. ellipticus) vs. *A. oerlinghausenensis* (ITS 99.4%, *tub*2 95.9%), and *A. westerdijkiae* vs. *A. ochraceus* (ITS 98.7%, *tub*2 94.7%).

The genus *Penicillium* was represented by 27 strains corresponding to four species (Supplementary Materials, Figure S2b): *P. crustosum* (section *Fasciculata*), *P. kongii* (section *Brevicompacta*), and *P. roqueforti* and *P. paneum* (section *Roquefortorum*). Species boundaries were primarily resolved through the use of *tub*2, given the high ITS identity among closely related taxa (99.6–99.8%). Clear separation was achieved between *P. roqueforti* and *P. mediterraneum*, *P. crustosum* and *P. solitum*, and *P. kongii* and *P. brevicompactum* (*tub*2, 97.0–99.1%).

The genus *Cladosporium* (Cladosporiaceae) was represented by four strains identified to three species (Supplementary Materials, Figure S2c), i.e., *C. cladosporioides* (*C. cladosporioides* complex) and *C. ramotenellum* and *C. limoniforme* (both members of the *C. herbarum* complex). Since ITS sequences were nearly identical among closely related taxa, species delimitation relied primarily on the use of *act* (pairwise identities 88.8–90.4%), with additional support from *tef*1-α and/or *tub*2.

*Candida boidinii* (Debaryomycetaceae) was the most frequently isolated yeast, since it was recovered from the majority of isolation treatments (Table 2). In the ITS + *tef*1-α phylogeny (Supplementary Materials, Figure S2d), the obtained strains grouped with the type strain (CBS 2428; [36]); however, they displayed a notable divergence (ITS, 96.8%; *tef*1-α, 95.2%), suggesting potential cryptic diversity. Multigene phylogeny in Saccharomycotina demonstrated that the genus *Candida* is an artificial and polyphyletic assemblage; this led to the transfer of *C. boidinii* and several taxa related to the genus *Ogataea* [38]. Nevertheless, this reclassification has not been universally adopted, and *C. boidinii* remains the most frequently cited name in applied microbiology, biotechnology, and environmental studies. For consistency with the prevailing literature and existing databases, we retain the traditional name *C. boidinii* in the present work.

Seven strains of *Geotrichum* (Dipodascaceae) were identified as *G. candidum,* despite demonstrating high ITS heterogeneity (92.9–99.5%). This taxon is currently treated as a single phylogenetic species, including strains identified as *G. bryndzae* and *G. silvicola,* which are considered synonyms (Supplementary Materials, Figure S2e) [39]. In addition, the ex-type strain CBS 772.71, formerly attributed to *G. candidum*, has been reassigned to *G. galactomycetum,* which also formed a distinct clade in our phylogenetic analysis (*G. galactomycetum* vs. *G. candidum*: 89.2–93.3% ITS identity). The recent taxonomic revision of the genus *Geotrichum* confirmed the complexity of *G. candidum* sensu lato, further highlighting the need for an exact assessment of the species boundaries.

Two strains were identified as *Pleurostoma richardsiae* (Pleurostomataceae) by using a five-marker concatenated phylogeny (ITS, 28S, *rpb*2, *tef*1-α, and *tub*2; Supplementary Materials, Figure S2f); one strain grouped with the isotype (CBS:270.33) and the ex-type of the synonym *Phialophora calyciformis* (strain A177 from type CBS 302.62), while the other clustered with a strain isolated from olive trees in Italy (P2BA), forming a well-supported subgroup. Despite high identities in ITS and 28S (>99.0%), the divergence noted in *tub*2 (95.2%), *rpb*2 (98.0%), and *tef*1-α (98.2%) suggests possible cryptic differentiation within the genus.

Three strains were assigned to *Talaromyces nanjingensis* (Trichocomaceae) based on ITS+*tub*2 phylogeny (Supplementary Materials, Figure S2g), while the *tef*1-α shows 99.7% identity to the respective part of the holotype’s (JP-NJ4) genome. This species, recently described from *Pinus* rhizosphere soil in China [40], was distinguished from its closest phylogenetic relatives (*T. lianis*, *T. brevis*) primarily by the *tub*2 marker (98.7% and 98.0% identity, respectively), while the ITS sequences were identical. This is the first report on the presence of *T. nanjingensis* in Europe and in a new habitat.

Several other taxa in TPOMW were each represented by a single isolate, i.e., *Barnettozyma californica* (Phaffomycetaceae), *Beauveria pseudobassiana* (Cordycipitaceae), *Sarocladium kiliense* (Sarocladiaceae), *Neocucurbitaria keratinophila* (Cucurbitariaceae), and *Stagonosporopsis ailanthicola* (Didymellaceae). Identification was mainly based on their ITS sequences, which were identical to the respective type material, i.e., CBS 252 (Supplementary Materials, Figure S2d), ARSEF 3405, CBS 122.29, MFLUCC 16-1439, and CBS 121759, respectively, while *tef*1-α sequences were used to further confirm their identity.

Especially as regards the genus *Trichoderma* (Hypocreaceae), two species new to science in the Harzianum clade were delineated and described herein as *Trichoderma amurcicola* and *T. olivarum*. Their delimitation was evidenced through the outcome of a multilocus phylogeny combining sequence data from ITS, *rpb2*, *tef1-α*, *cal*, and *act* markers (Figure 5), and was further supported by distinct morphological and cultural characters.

*T. amurcicola* formed a moderately supported clade (MLBS: 66%) with *T. simile* [41], nested within a broader, strongly supported group (MLBS: 98%; BPP: 1.00) also including *T. guizhouense* [42], *T. pholiotae* [43], and two undescribed lineages (*T.* cf. *guizhouense* #1, #2). Despite high ITS (99.6% vs. *T. simile*, 98.9% vs. *T. guizhouense*) and *tef*1-α (≥99.6%) identities with closely related strains, clear separation was provided by *rpb*2, which showed 1.6–3.5% divergence (98.3–98.4% vs. *T. simile*, 97.9–98.0% vs. *T. guizhouense*, 96.5–96.7% vs. *T.* cf. *guizhouense* #1). Additional support was provided through the use of the *cal* (99.1% vs. *T. guizhouense*, 99.1–99.3% vs. *T.* cf. *guizhouense* #1) and *act* markers (98.8% vs. *T. guizhouense*, 98.9–99.7% vs. *T.* cf. *guizhouense* #1). These values are below commonly accepted species-level thresholds in the genus *Trichoderma* [44,45].

It should be noted that several strains previously identified as *T. guizhouense*, including genome-sequenced or reference strains such as NJAU 4742 and GJS 97-28 from Japan [46], grouped outside the clade, which included the species holotype HGUP0038 from China [42,47], indicating taxonomic discrepancies. These strains were recovered within distinct, well-supported clades corresponding to *T.* cf. *guizhouense* #1 and #2, with BAFC 4370 and BAFC 4356 [46] serving as representative vouchers, respectively. In the aforementioned study, GJS 97-28 was erroneously designated as an ex-type strain of *T. guizhouense*, despite its clear phylogenetic incongruence and distant geographic origin from the species’ type locality. Furthermore, one Croatian isolate (S278) [44], formerly misassigned to *T. guizhouense*, was grouped into *T. amurcicola*. Additional isolates annotated as “*T. guizhouense*”, including both published (e.g., S628 from Greece; [44]) and unpublished sequences (e.g., Tr49 and Tr118 from Turkey; SZMC:1630, SZMC:1633 from Hungary; SZMC 25242, SZMC 25749 from Serbia; SZMC:12334, SZMC:12384 from India; PARC1022, PARC1023, PARC1025, and PARC1026 possibly from southern Italy) also grouped outside the true *T. guizhouense* clade and within *T. amurcicola* (according to *rpb*2 BLASTn results and phylogenetic analysis), thus suggesting a wider Eurasian distribution of the *T. amurcicola*. Although these strains were often considered to represent a single taxonomic entity (*T. guizhouense*), previous phylogenetic analyses frequently failed to group them within a monophyletic, well-supported clade [48], highlighting the need for multigene approaches in species delimitation within the Harzianum clade and the critical role of holotype-based validation/identification.

*T. olivarum* was resolved as a sister to *T. simmonsii* (MLBS: 99%, BPP: 1.00). Although the two species exhibited high ITS and *tef*1-α identity (100% and 99.6%, respectively), clear separation was evident through the use of the other markers used, i.e., *rpb*2 (97.8%), *cal* (95.7%), and *act* (98.9%). These values are below established species-level thresholds, and when combined, *T. olivarum* is clearly separated as a distinct lineage within the Harzianum clade. Interestingly, despite their phylogenetic distance, *T. olivarum* and *T. subvermifimicola* shared identical *rpb*2 sequences, demonstrating the limitations of single-locus resolution in species delimitation within the genus *Trichoderma*. In addition, the sequence from the genome of an isolate from South Korea (GH-Sj1, initially labeled *T. simmonsii*), grouped outside the *T. simmonsii* clade, which includes the holotype, thus representing a different taxon, which is provisionally named *T.* cf. *simmonsii*.

#### Taxonomy

**Figure 6:**
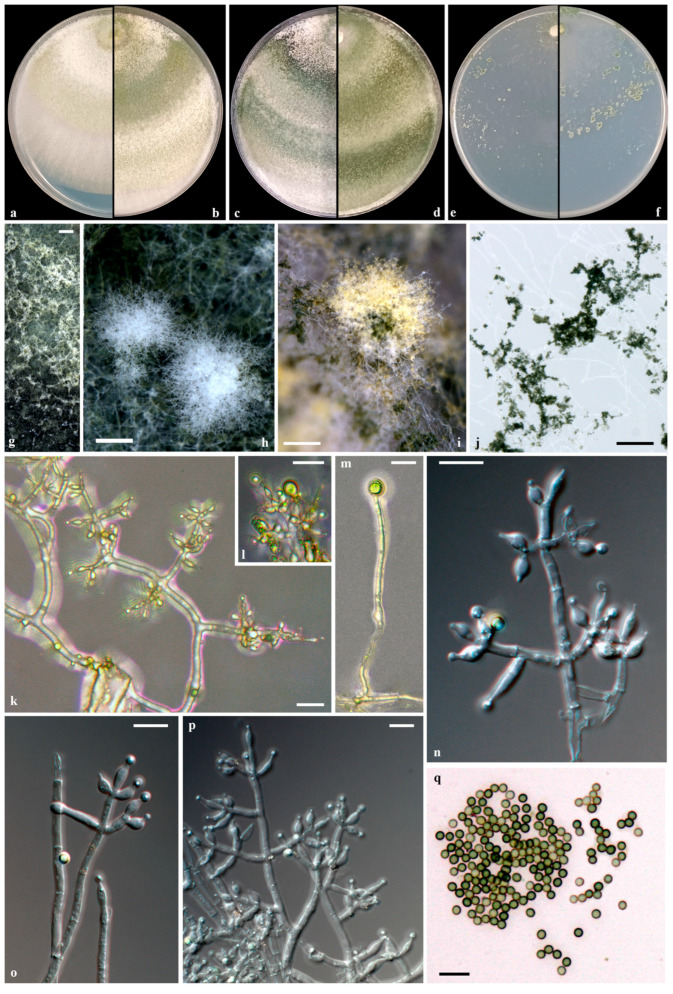
*Trichoderma amurcicola* sp. nov. (LGAM SOW_MF2a). Cultures on PDA at 25 °C after 3, 5, and 7 days ((**a**–**c**), respectively), at 30 °C after 7 days (**d**), and on SNA at 25 °C after 7 days (**e**,**f**); conidiation: aerial hyphae and pustules on PDA (**g**–**i**) and on SNA (**j**); conidiophores and phialides (**k**,**l**,**n**–**p**); chlamydospores (**l**,**m**); conidia (**q**). Scale bars: (**g**–**j**) 0.25 mm, (**k**–**q**) 10 μm.

***Trichoderma amurcicola*** **V. Fryssouli, I. Kefalogianni, E. Polemis & G.I. Zervakis, sp. nov.** **(**Figure 6**)**

**Mycobank:** MB860058.

**Etymology**: “amurcicola” from the Latin ‘amurca’ (deriving from Greek ἄμουργα), meaning the olive pomace or the sediment/residue remaining after olive oil extraction), and from the suffix ‘-cola’ (meaning residing/existing in Latin), referring to the substrate from which the fungus was isolated.

**Diagnosis:** *Trichoderma amurcicola* is distinguished within the Harzianum clade by the multilocus phylogeny (ITS, *tef*1-α, *rpb*2, *cal*, *act*), the relatively narrow phialides, the presence of rare terminal chlamydospores, and the presence of diffusible pigments.

**Type**: GREECE, Peloponnese, from TPOMW, November 2017. Holotype LGAM SOW_MF2a, preserved in 30% (*v*/*v*) glycerol at −80 °C in the Laboratory of General and Agricultural Microbiology, Agricultural University of Athens (Greece). GenBank accession numbers: ITS = PP766637, *cal* = PP768730, *act* = PP768723, *rpb*2 = PP768717, *tef*1-α = PP768704.

**Conidiophores** hyaline, smooth-walled, pyramidal to tree-like, typically with 1–3 levels of verticillate branching, forming a densely intricate reticulum. Main axis rebranch, producing paired, opposing, or singly side-branches that emerge predominantly asymmetrically and perpendicular to the axis or slightly oriented toward the terminus. Branch intervals measure (2.4-)7.0-25.1(-57.5) μm, mean 15.2 μm; septa conspicuous. Branches terminating in cruciate whorls of 2-4 (-5) phialides, occasionally solitary. **Phialides** predominately ampulliform to lageniform, straight to curved or inequilateral, distinctly constricted below the apex, forming symmetrical or slightly curved narrow necks, (4.6-)5.0-10.2(-11.7) × (2.1-)2.3-3.4(-3.6) μm, mean 7.3 × 2.9 μm, l/w (1.5-)1.7-3.8(-4.5), distinct base (0.9-)1.2-2.2(-2.4) μm wide; supporting cell (1.8-)1.9-4.4(-5.5) μm wide (mean 2.4 μm); ratio of phialide length to supporting cell width (1.8-)2.0-4.5 and phialide width to supporting cell width (0.8-)0.9-1.6(-1.9). **Conidia** globose to subglobose, occasionally ellipsoid, smooth, thin-walled, hyaline with yellow-green to dark green hue, (2.5-)2.8-3.3(-3.5) × (2.1-)2.3-2.9(-3.1) μm, mean 3.0 × 2.5 μm, l/w (1.0-)1.1-1.3(-1.5). **Chlamydospores** occasionally observed in PDA, terminal, ellipsoid to globose, 5.4–6.0 × 3.4–5.8 μm, l/w 1.0–1.7.

On **PDA** after 72 h, colonies attain radii of 64–70 mm at 25 °C, 67–77 mm at 30 °C, and 38–41 mm at 35 °C, covering the plate in 3 days at 25 °C. Colonies exhibit radial growth with dense, continuous cottony to floccose aerial mycelium, especially near inoculum; margin rather flat and well-defined, initially white, developing broad concentric, not well-defined zones altering in color from yellowish green, greyish green to olive-green, darkening with age; dark green in the center. Conidiation initiates within 48 h on aerial hypha, and minute pustules are formed around the inoculum and margin, transitioning from white to dark green in the center. Reverse yellow to orange diffuse pigmentation. Odor faintly fruity.

On **SNA** after 72 h, colony radii measure 37–43 mm at 25 °C, 30–43 mm at 30 °C, and 13–15 mm at 35 °C, covering the plate in 5 days at 25 °C. Colonies translucent, thin, and radially structured, with sparse, arachnoid aerial hyphae in concentric zones; margin not well-defined. Conidiation occurs on both aerial hyphae and pulvinate pustules form within 48 h; pustules distributed either diffusely or in concentric arrangements, transitioning from white to dark green in the center. No diffuse pigmentation on the reverse side. Odor indistinct.

**Additional material examined**. GREECE, Peloponnese, from TPOMW, November 2017. LGAM SOW_MF2b, preserved in 30% (*v*/*v*) glycerol at −80 °C in the Laboratory of General and Agricultural Microbiology, Agricultural University of Athens (Greece). GenBank accession numbers: ITS = PP766638, *cal* = PP768731, *act* = PP768724, *rpb*2 = PP768718, *tef*1-α = PP768705.

**Notes**. *Trichoderma amurcicola* is phylogenetically closely related to *T. simile* [41] and *T. guizhouense* [42,47], yet it is readily distinguishable based on consistent morphological and cultural characteristics. All three species exhibit pyramidal to tree-like conidiophores; however, they differ diagnostically in phialide morphology. The phialides of *T. amurcicola* (5.0-10.2 × 2.3-3.4 μm; l/w 1.7-3.8) are narrower and less variable than those of *T. simile* (4.3-11.9 × 2.7-3.9 μm; l/w 1.3-4.4). In contrast, *T. guizhouense* displays broader and shorter phialides (4.7-7.5 × 3.0-3.7 μm; l/w 1.4-2.4) according to Chaverri et al. [47], or rather narrow phialides (4.5-10 × 2-3 μm) according to Li et al. [42], since the two available descriptions differ notably in measurements. Conidial morphology (size and shape) does not provide reliable differentiation among these species. *T. amurcicola* produces globose to subglobose conidia (2.8-3.3 × 2.3-2.9 μm; l/w 1.1-1.3), overlapping with the slightly smaller oval conidia of *T. simile* (2.6-3.2 × 2.2-2.8 μm; l/w 1.0-1.2), whose low l/w ratio values more closely resemble globose forms. *T. guizhouense* produces primarily globose conidia (2-3 μm in diameter; 42; or 2.5-3.2 × 2.5-3.0 μm; l/w 1.0-1.2; 47). Chlamydospores in *T. amurcicola* are rare, exclusively terminal, and measure 5.4-6.0 × 3.4-5.8 μm (l/w 1.0-1.7). In contrast, *T. simile* forms abundant chlamydospores, both terminal and intercalary, and ranges in size (4.2-7.8 × 4.0-7.2 μm), whereas *T. guizhouense* lacks chlamydospores [42,47].

In culture, *T. amurcicola* exhibits faster growth at 35 °C (when measured for 72 h), particularly on PDA (38–41 mm), compared to *T. simile* (10 mm) and *T. guizhouense* (16–20 mm; 42), and similarly on SNA (13–15 mm vs. *T. simile*: 3 mm; *T. guizhouense*: 5–8 mm; 42). However, at 25 °C (for 72 h) on SNA, *T. amurcicola* grows more slowly (37–43 mm) than *T. simile* (47 mm) and *T. guizhouense* (58–61 mm; 42). Colonies of *T. amurcicola* produce yellow to orange diffusible pigments on PDA and emit a faint fruity odor, i.e., traits shared with *T. guizhouense* [42,47], but absent in *T. simile*.

In comparisons with more distantly related taxa, *T. pholiotae* [43] is readily differentiated by its larger and broader phialides (4.9-10.9 × 2.4-4.2 μm; l/w 1.4-3.4) and conidia (2.6-3.8 × 2.4-3.3 μm; l/w 1.0-1.3), as well as the frequent occurrence of larger terminal and intercalary chlamydospores (5.0-7.4 × 4.9-7.0 μm). *T. asiaticum* [41] can be distinguished by its considerably shorter phialides (4.0-6.0 × 2.0-3.0 μm; l/w 1.3-3.0), smaller conidia (2.4-3.0 × 2.1-2.7 μm; l/w 1.1-1.3), and the absence of chlamydospores. *T. pseudoasiaticum* [41] also forms slightly broader phialides (6.1-9.0 × 2.6-3.6 μm; l/w 1.5-3.6) and nearly spherical conidia (2.4-3.2 × 2.4-3.0 μm; l/w 1.0–1.1), while terminal chlamydospores are frequently observed (4.7-7.7 × 4.0-7.6 μm). None of these three species produce diffusible pigments, and none exhibit fast growth under thermotolerant conditions (35 °C for 72 h) on SNA (8–10 mm for *T. pholiotae*, 7 mm for *T. asiaticum*, and 2 mm for *T. pseudoasiaticum*).

**Figure 7:**
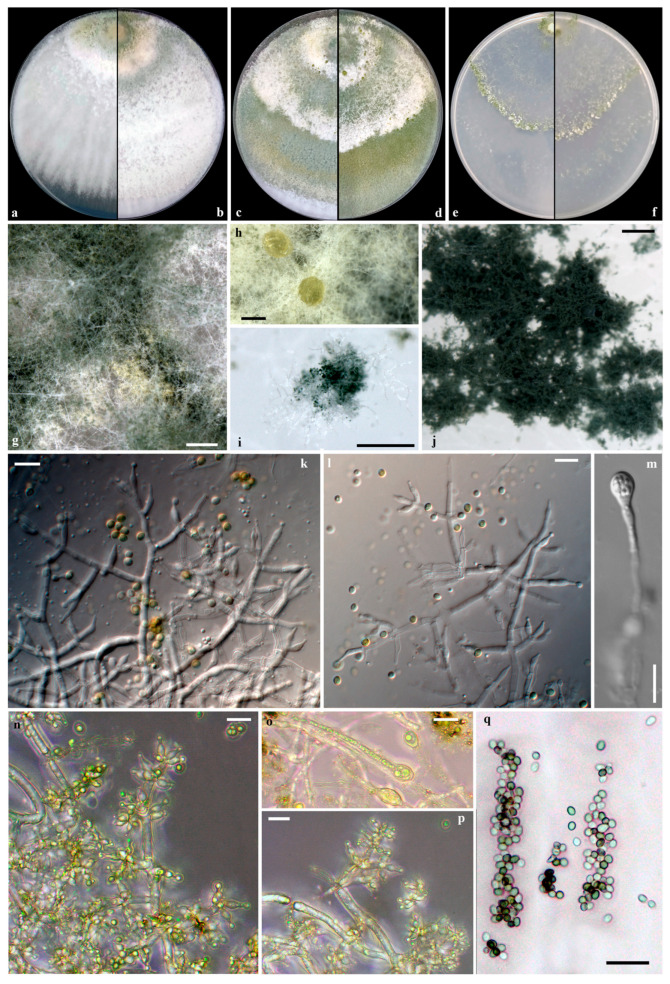
*Trichoderma olivarum* sp. nov. (LGAM SOW_MF1a). Cultures on PDA at 25 °C after 3, 5, and 7 days ((**a**–**c**), respectively), at 30 °C after 7 days (**d**), and on SNA after 7 days at 25 °C and 30 °C ((**e**,**f**), respectively); conidiation: aerial hyphae and pustules on PDA (**g**,**h**) and on SNA (**i**,**j**); conidiophores and phialides (**k**,**l**,**n**,**p**); chlamydospores (**m**,**o**); conidia (**q**). Scale bars: (**g**–**j**) 0.25 mm, (**k**–**q**) 10 μm.

***Trichoderma olivarum*** **V. Fryssouli, E. Polemis, Μ.A. Typas & G.I. Zervakis, sp. nov.** **(**Figure 7**)**

**Mycobank:** MB860059.

**Etymology**. “olivarum” from the Latin ‘olivarum’ (genitive plural of ‘olivarium’, meaning olive grove), denoting the olive-associated habitat from which this fungus originated.

**Diagnosis**. *Trichoderma olivarum* is distinguished within the Harzianum clade by the multilocus phylogeny (ITS, *tef*1-α, *rpb*2, *cal*, *act*), the relatively long and narrow phialides, the often ovoid to oblong conidia, and the frequently present chlamydospores.

**Type**. GREECE, Peloponnese, from TPOMW, Nov. 2017. Holotype LGAM SOW_MF1a, preserved in 30% (*v*/*v*) glycerol at −80 °C in the Laboratory of General and Agricultural Microbiology, Agricultural University of Athens (Greece). GenBank accession numbers: ITS = PP766635, *cal =* PP768728, *act =* PP768721, *rpb*2 *=* PP768715, *tef*1-α *=* PP768702.

**Conidiophores** hyaline, smooth-walled, pyramidal to tree-like, typically with 1–2(–3) perpendicular rebranching levels, forming a densely intricate reticulum where the main axis is often unrecognizable. Main axis producing single or paired opposing side-branches that emerge asymmetrically. Branch intervals measure (2.5-)4.2-27.8(-30.3) μm, mean 15.2 μm; septa conspicuous. Branches terminating in whorls of 2–4 phialides or solitary. **Phialides** ampulliform to lageniform, straight to curved or inequilateral, distinctly constricted below the apex, forming symmetrical or slightly bent narrow necks usually elongated, especially in the terminal solitary phialides, (5.4-)5.7-12.4(-13.1) × (1.9-)2.1-3.1(-3.3) μm, mean 8.7 × 2.6 μm, l/w 1.7-6.0, distinct base, 0.9–2.4 μm wide (mean 1.7 μm); supporting cells 1.9–3.1 μm wide (mean 2.4 μm); ratio of phialide length to supporting cell width 1.9–5.7 (mean 3.6), and phialide width to supporting cell width 0.7–1.4 (mean 1.1). **Conidia** globose, subglobose, ovoid to oblong, smooth, thin-walled, hyaline with pale green, yellow-green to dark green hue, (2.8-)2.9-3.9(-4.2) × (2.1-)2.3-2.9(-3.0) μm, mean 3.4 × 2.6 μm, l/w (1.0-)1.1-1.5(-1.9). **Chlamydospores** frequently present, terminal to intercalary, globose to ellipsoid, 5.6-9.6 × 5.1-6.5 μm, l/w 1.0-1.5.

On **PDA** after 72 h, colonies attain radii of 69–73 mm at 25 °C, 73–74 at 30 °C, 17–28 mm at 35 °C, covering the plate in 3 days at 25 °C. Colonies radially structured, with dense, continuous cottony to floccose aerial mycelium, especially near inoculum, initially white transitioning to dark green with age, developing broad concentric zones, white to olivaceus; margin rather flat and inconspicuous with faint mycelium. Conidiation initiates within 48 h on aerial hypha; pustules are formed around the inoculum and margin, transitioning from white to dark green in the center. No diffuse pigmentation on the reverse side. Odor faintly fruity.

On **SNA** after 72 h, colony radii measure 47–49 mm at 25 °C, 51–55 mm at 30 °C, and 5–7 mm at 35 °C, covering the plate in 5 days at 25 °C. Colonies translucent, thin, and radially structured, with sparse, arachnoid aerial hyphae concentrated on concentric zones; margin not well-defined. Conidiation occurs on pulvinate, compact pustules within 48 h, with pustules distributed near the inoculum and in concentric arrangements, transitioning from white to dark green in the center. No diffuse pigmentation on the reverse side. No distinctive odor is noted.

**Additional material examined**. GREECE, Peloponnese, from TPOMW, Nov. 2017. Holotype LGAM SOW_MF1b, preserved in 30% (*v*/*v*) glycerol at −80 °C in the Laboratory of General and Agricultural Microbiology, Agricultural University of Athens (Greece). GenBank accession numbers: ITS = PP766636, *cal =* PP768729, *act =* PP768722, *rpb*2 *=* PP768716, *tef*1-α *=* PP768703.

**Notes**. Detailed comparisons of morphological, physiological, and culture features reveal clear species-level distinction of *T. olivarum* from its sister species, *T. simmonsii* [47], and also from *T. subvermifimicola* [48], which present identical *rpb*2 sequences despite their relative distant topology and divergence demonstrated when using other markers. The three species share broadly similar conidiophore architecture (pyramidal, asymmetrically branched at 1–3 levels) similar to *T. simmonsii*, whereas *T. subvermifimicola* shows more symmetrical pyramidal arrangements. Phialide morphology is a key diagnostic character. In *T. olivarum*, phialides are longer and narrower (5.7-12.4 × 2.1-3.1 μm; l/w 1.7-6.0), contrasting with the shorter, broader phialides of *T. simmonsii* (5.2-6.5 × 3.0-3.7 μm; l/w 1.5-2.4) and of *T. subvermifimicola* (4.7-9.4 × 2.3-3.6 μm; l/w 1.4-3.9). Conidial dimensions also support species delimitation: *T. olivarum* forms variable, globose to oblong conidia (2.9-3.9 × 2.3-2.9 μm; l/w 1.1-.5), larger and more elongate than those of *T. simmonsii* (2.7-3.2 × 2.5-3.0 μm; l/w 1.0-1.1) and *T. subvermifimicola* (2.7-3.3 × 2.5-3.0 μm; l/w 1.0-1.2). In addition, *T. olivarum* forms frequent, large terminal and intercalary chlamydospores (5.6-9.6 × 5.1-6.5 μm), whereas *T. simmonsii* produces them rarely, and *T. subvermifimicola* forms smaller (4.0-6.9 × 3.5-6.4 μm), also intercalary and terminal chlamydospores.

Colony growth characteristics further distinguish *T. olivarum* from related species. On PDA at 25 °C, it reaches 69–73 mm in 72 h, outgrowing *T. simmonsii* (55–65 mm) and *T. vermifimicola* (60–64 mm), while *T. simmonsii* exhibits better growth at 35 °C (25–55 mm vs. 17–28 mm in *T. olivarum*, and 13–17 mm in *T. subvermifimicola*). Colonies do not produce diffuse pigmentation; however, *T. simmonsii* and *T. subvermifimicola* sometimes produce yellow pigmentation on the reverse surface.

### 3.2. Preliminary Evaluation of Biodegradation Efficacies of Isolated Fungi

In the frame of performing a preliminary evaluation of the biodegradation potential of the isolated fungi, 66 strains corresponding to 31 species were tested with respect to their hydrolytic, oxidative, and dye-decolorizing efficacies. The enzymatic index (EI) was assessed on cellulose, xylan, lignin, guaiacol, and RBBR substrates (Table 2; Supplementary Materials, Table S10). Τhe tested strains exhibited high degradation potential; 96% of them displayed cellulolytic activity, and 82% were capable of degrading xylan (22% and 19% were evaluated as significant degraders). In contrast, ligninolytic activity and laccase production were observed in fewer species (i.e., 13% and 17%, respectively), suggesting specialization in oxidative mechanisms for certain taxa. In addition, 72% of the strains displayed the ability to decolorize RBBR (5% were evaluated with strong activity), indicating a significant prevalence of dye-degrading capabilities within the collection. Despite variable enzymatic activity, all strains were able to grow well on lignocellulosic media.

As regards cellulose degradation, the highest cellulolytic activities were exhibited by *Aspergillus sydowii*, *Fuscoporia ferrea*, *Penicillium paneum*, *P. crustosum*, and *Beauveria pseudobassiana* strains. In addition, *Aspergillus novoparasiticus*, *Penicillium kongii*, *Pleurostoma richardsiae,* and *Trichoderma amurcicola*, as well as all strains of Mucorales (*M. circinelloides*, *M. pseudolusitanicus*, *M. racemosus*) consistently displayed strong cellulolytic profiles. As concerns xylan degradation, the highest xylanolytic activity was detected in *Beauveria pseudobassiana*, *Geotrichum candidum*, *Peniophora lycii*, and *Neocucurbitaria keratinophila*. Notable xylan-degrading capabilities were also detected in *Mucor circinelloides* and *M. pseudolusitanicus*, the two new *Trichoderma* species, and in several *Aspergillus* and *Penicillium* strains. Ligninolytic activity was most pronounced in the basidiomycetes *Pseudophlebia setulosa*, *Fuscoporia ferrea*, and *Peniophora lycii*. Among Ascomycota, *Cladosporium ramotenellum* and *C. limoniforme* exhibited the strongest lignin-degrading and laccase activities, while *Pleurostoma richardsiae* and *Beauveria pseudobassiana* were also efficient in this respect. In contrast, *Aspergillus fumigatus* and *A. filifer* grew well on lignin-containing media but did not exhibit detectable ligninolytic activity. Strong RBBR decolorization, indicative of oxidative potential, was observed in *Beauveria pseudobassiana* and *Candida boidinii*. Additional high decolorization activities were noted in *Geotrichum candidum*, *Barnettozyma californica*, *Cladosporium limoniforme*, and *C. ramotenellum*. Moderate dye-degrading capacity was widespread across species of Basidiomycota, and of the genera *Aspergillus*, *Penicillium*, and *Mucor*.

More importantly, a subset of isolates exhibited broad enzymatic repertoires across all substrates tested. These included *Cladosporium ramotenellum*, *C. limoniforme*, *Fuscoporia ferrea*, and *Pseudophlebia setulosa*, while significant activities were also detected in *Beauveria pseudobassiana*, *Trichoderma amurcicola*, *Pleurostoma richardsiae*, and *Peniophora lycii*. In addition, several taxa already known from the literature for their broad biotechnological relevance—including *Aspergillus sydowii*, *Barnettozyma californica*, *Candida boidinii*, *Geotrichum candidum*, and *Mucor circinelloides*—also displayed strong and versatile enzymatic profiles.

## 4. Discussion

### 4.1. Fungal Diversity in TPOMW

The culturable fungal community in TPOMW was dominated by Ascomycota, particularly Eurotiales and Hypocreales, with Basidiomycota and Mucoromycota recovering at lower numbers, which is in accordance with previous findings [4,15,22]. This pattern highlights the selective ecological pressures imposed by such types of substrates, i.e., with acidic pH, high content in lipids, phenolics, and other chemical stressors [1]. TPOMW exerts a strong selective effect on fungal communities, explaining the frequent recovery of *Aspergillus*, *Cladosporium*, *Mucor*, and *Penicillium* strains as previously noted [15,16,21]. These taxa share common ecological strategies favoring their growth and persistence under environmentally challenging conditions, including rapid growth, tolerance to phenolic compounds, and the capacity to exploit complex carbon sources. In addition to filamentous taxa, *Candida* and yeast-like *Geotrichum* strains were abundant, reflecting their ability to thrive in sugar- and lipid-rich niches and to colonize substrates at early stages of decomposition [15,19,20]. Their recurrent presence across diverse agro-wastes suggests convergent ecological strategies shaped by nutrient-rich but chemically restrictive environments. Furthermore, two new *Trichoderma* species (*T. amurcicola* and *T. olivarum*) were also found in TPOMW, indicating that this substrate could serve as a reservoir of unexplored fungal diversity.

Basidiomycetes are less frequently isolated from OMW-based substrates; the genera *Pseudophlebia*, *Fuscoporia*, and *Peniophora*, which are typically associated with wood degradation, are recorded for the first time in TPOMW. This was also the case for *Beauveria*, *Neocucurbitaria*, *Pleurostoma,* and *Stagonosporopsis*, which are mostly known as pathogens of plants, insects, or animals [49,50,51]; their presence in TPOMW reflects their wide adaptation in different ecological niches. In addition, *Barnettozyma californica*, *Cladosporium limoniforme*, *Mucor pseudolusitanicus*, and *Talaromyces nanjingensis* were recorded for the first time in olive mill wastes.

These patterns underline the strong selection effect of TPOMW on fungal communities and reveal a structured mycobiota composed of stress-tolerant filamentous fungi (mainly Ascomycota and Mucoromycota), opportunistic yeasts, and (occasionally) filamentous basidiomycetes [4,15,22]. This diversity provides an ecological basis for understanding fungal succession in phenolic- and lipid-rich agro-industrial residues.

### 4.2. General Taxonomic and Phylogenetic Remarks on the Genus Trichoderma

Our analyses outlined the widespread taxonomic issues related to type collections and genome-linked strains in the genus *Trichoderma*. Beyond the case of the type material of *T. guizhouense*, which was previously discussed, additional issues were identified in the course of our investigation. One of them relates to *T. afarasin* collections reported as type material in different studies, i.e., CBS 130755 by Chaverri et al. [47] and Dis 314f by Barrera et al. [46]. Further confusion arises when the identity of a strain has changed over time without the respective necessary updates in the INSDC databases. For example, sequences derived from the holotypes of *T. inhamatum* (CBS 273.78; FJ577683) and *T. endophyticum* (CBS 130729/DIS 217A; FJ442243, FJ463319, FJ442292) remain deposited under the name “*T. harzianum*”, while the sequences derived from the holotype of *T. syagri* are still labeled as *T. camerunense* (BAFC 4357; MG822711, MG822714, MG822717). Moreover, some strains are linked to multiple collection codes, complicating accurate traceability and material management (Table S10). An indicative example is the type of *T. austroindianum* (i.e., strain BAFC 3583 [46]), which in NCBI appears under VAB-T050 without any reference to the former code and without mentioning that it corresponds to the type material.

Inconsistencies were also detected as regards the association of genome sequences with validated species concepts. Several genome sequences were found to be misassigned, appearing in clades not related to their taxonomic identity. For example, strain CFAM-422 (submitted as *T. lentiforme*) clusters within the *T. neotropicale* lineage, strain MUT 3171 labeled as *T. lixii* groups within the *T. harzianum* clade, and strain FJ059 identified as *T. semiorbis* is phylogenetically placed within the *T. rugulosum* group. Another notable case involves the genome of the type strain of *T. brevicrassum* (TC967) [52], which in our work nested within the clade, which includes the *T. breve* holotype. This is in contrast with their phylogenetic placement in the study where both species were originally described [53]; the former was placed in the Chlorosporum clade, whereas the latter forms part of the Harzianum clade. This discordance indicates a probable misidentification and calls for genomic re-evaluation of the deposited sequence. Furthermore, the type strain CBS 226.95 of *T. harzianum* also displays conflicting placements. Based on sequence data from Chaverri et al. [47], it clusters—as expected—within the *T. harzianum* clade, and this placement is congruent with published phylogenies. However, the genome assigned to CBS 226.95 (unpublished) falls into a separate clade, herein provisionally designated as *T.* cf. *harzianum*.

These findings reveal a series of pressing issues in the *Trichoderma* taxonomy and nomenclature. Persistent taxonomic gaps such as misassigned genomes and ambiguous type designations continue to undermine species-level resolution. Furthermore, our results confirm that *rpb*2 and *cal* were the most informative markers for species delimitation within the Harzianum clade, offering higher resolution compared to ITS and *tef*1-α. In particular, *rpb*2 showed consistency in delimiting among species (with some exceptions like the low resolution between *T. olivarum* and *T. subvermifimicola*) and a clear barcoding gap across comparisons, confirming previous findings on its discriminatory power in *Trichoderma* [45]. Despite their usefulness, *cal* and *act* were less broadly available in public databases, limiting their comparative value. *tef*1-α resolution was rather inconsistent, which is mostly due to the different target regions used among studies [47,54].

Consistent morphological differences (e.g., phialide and conidial dimensions, chlamydospore morphology) and physiological traits (e.g., growth at higher temperatures) further contribute to species separation. These findings support the necessity of a polyphasic taxonomic approach, especially for cryptic taxa.

### 4.3. Biodegradation Potential of Fungal Strains

The enzymatic profiles of TPOMW-associated fungi highlight the coexistence of distinct functional guilds that play complementary roles in substrate degradation. Filamentous Ascomycota, including *Aspergillus*, *Cladosporium, Penicillium*, *Talaromyces*, and *Trichoderma,* exhibited broad hydrolytic and, in several cases, ligninolytic capacities, as previously demonstrated [12,15,21]. These features are consistent with their frequent dominance in lipid-, phenolic-, and lignocellulose-rich environments, where rapid growth, metabolic versatility, and tolerance to chemical stressors provide a competitive advantage [55,56,57,58,59,60,61,62,63]. Within this group, thermotolerant isolates such as *Aspergillus fumigatus* and *Trichoderma amurcicola* are of particular interest since their ability to function at elevated temperatures aligns with potential applications in biomass pretreatment and other industrial and biotechnological processes [11]. This is also reported in the past for *Aspergillus fumigatus* [64,65,66,67], *A. tubingensis* [68], and *A. welwitschiae* [69,70,71], although the respective strains isolated from TPOMW did not show a high enzymatic potential. Species of *Cladosporium,* though less frequently reported in pertinent studies [61,72], demonstrated significant ligninolytic activities, indicating their potential exploitation in the degradation of recalcitrant substrates.

The presence of *Barnettozyma*, *Candida,* and *Geotrichum* yeasts reflects their capacity to colonize sugar- and lipid-rich substrates, and to initiate early stages of substrate decomposition [20]. Their enzymatic versatility, including hydrolytic and oxidative dye-degrading activities, is consistent with their reported applications in bioethanol production, wastewater detoxification, value-added metabolite synthesis, or broader interest [73,74,75,76,77,78,79]. White-rot basidiomycetes (*Pseudophlebia*, *Fuscoporia*, *Peniophora*), though isolated less frequently, exhibited high ligninolytic and laccase activities, confirming their ecological relevance and biotechnological potential in the degradation of lignocellulosics [10,80,81,82,83].

Members of Mucorales, especially *Mucor circinelloides*, displayed notable hydrolytic activity, dye-decolorization capacity and rapid substrate colonization. These features underline their ecological role as early colonizers in shaping fungal succession and driving initial biomass conversion. Furthermore, several strains already examined in applications related to bioethanol production, lipid accumulation, and enzyme secretion [84,85,86,87,88].

As regards other less common—but enzymatically active—fungi isolated from TPOMW, *Beauveria pseudobassiana* had been primarily studied for use in agriculture as an insect pathogen; however, it revealed strong hydrolytic and ligninolytic activities, suggesting broader biotechnological potential [89,90]. In addition, *Pleurostoma*, *Neocucurbitaria,* and *Sarocladium*, usually investigated as plant or human pathogens [49,50], exhibited hydrolytic and dye-decolorizing activities that expand their ecological significance and exploitation potential [22,60].

Preliminary assays revealed widespread cellulolytic and xylanolytic activity, with substantial dye decolorization and targeted ligninolytic potential in Basidiomycota and Dothideomycetes. Strains from several species (i.e., *Beauveria pseudobassiana*, *Cladosporium ramotenellum*, *C. limoniforme*, *Fuscoporia ferrea,* and *Pseudophlebia setulosa*) exhibited a broad spectrum of enzymatic activities across all tested substrates. Additional ‘multifunctional’ strains were those of *Mucor circinelloides*, *Aspergillus sydowii*, *Candida boidinii*, and *Geotrichum candidum* with potential applications in biofuel production, enzyme biocatalysis, and agricultural biotechnology, as was previously evidenced for these particular species [55,73,74,77,78,79,84,86]. The importance of targeting culturable fungi, which remain accessible for downstream applications, is apparent. Further research should focus on optimizing microbial/fungal consortia (e.g., thermotolerant species/strains, yeasts) [14,59,65,68,76], enzyme production conditions, and scaling up of bioprocesses to fully harness their potential in sustainable waste management and circular bioeconomy systems.

## 5. Conclusions

This study contributes to the assessment of the taxonomically and functionally diverse culturable mycobiota existing in TPOMW, which is mainly shaped by the selective pressure of this chemically complex substrate. Beyond recovering well-known genera such as *Aspergillus*, *Penicillium*, and *Candida*, we documented previously unreported taxa in olive mill wastes, including two new species of *Trichoderma* (i.e., *T. amurcicola* and *T. olivarum*). Preliminary enzymatic screening revealed broad hydrolytic, ligninolytic, and dye-decolorizing activities, highlighting the presence of multifunctional strains with potential applications in biorefineries, biofuel production, wastewater treatment, and sustainable agriculture. By combining multigene phylogenetics with functional assays, this work evidences that TPOMW—albeit overlooked—could serve as a valuable reservoir of fungal diversity, including strains of particular biotechnological relevance. While culture-dependent methods and preliminary enzymatic assays do not reveal the full potential of the organisms present in TPOMW, they provide an effective entry point for identifying promising strains. Future research could focus on determining the enzymatic potential of isolated strains through quantitative assays, the establishment of potent fungal consortia, and their exploitation in biotechnological applications.

## Figures and Tables

**Table 1:** Identity of fungal isolates recovered from TPOMW, including species names, strain codes, number of MOTUs, and GenBank accession numbers for ITS, *tef*1-α, *tub*2, *rpb*2, *act,* and *cal* sequences produced in this work. The only 28S sequence generated in this study had the accession number PP748919 and was obtained from *Pleurostoma richardsiae* LGAM SOW_M3. Species are presented in alphabetical order per phylum, then family, and finally genus. Strain codes are provided by omitting the common-to-all prefix “LGAM SOW_”.

Species	Strain Code	No. of MOTUs	ITS	*tef*1-α	*tub*2	*rpb*2	*act*	*cal*
ASCOMYCOTA								
*Aspergillus* (Aspergillaceae)								
*A. filifer*	A5	5	PP766592		PP768675			
*A. fumigatus*	A1	3	PP766593		PP768676			
	A2	3	PP766594		PP768677			
	A3	3	PP766595	PP768693	PP768678			
*A. novoparasiticus*	T1	1	PP766596		PP768679			
	T2	1	PP766597					
*A. sydowii*	PT1b	4		PP768694	PP768680			
*A. tubingensis*	A6	7	PP766598					
	A7	7	PP766599		PP768681			
	PT4a	7	PP766600					
*A. welwitschiae*	A4	6	PP766601					
	A8	6	PP766602		PP768682			
	PT1	6	PP766603	PP768695				
*A. westerdijkiae*	A9	2	PP766604					
*Penicillium* (Aspergillaceae)								
*P. crustosum*	M1b	9	PP766605	PP768696				
	M2	10	PP766606					
	M7	10	PP766607	PP768697				
	M6	11	PP766608		PP768683			
	P1	11	PP766609					
	P2	12	PP766610					
	P3	12	PP766611					
	P4	13	PP766612					
	P5	13	PP766613					
*P. kongii*	PT2	8	PP766614					
	PT5	8	PP766615					
*P. paneum*	M8	14	PP766616	PP768698				
	M15	14	PP766617					
*P. roqueforti*	M5	15	PP766618	PP768699	PP768684			
	M12	15	PP766619	PP768700				
	M16	16	PP766620					
	M16a	16	PP766621					
	M16b	16	PP766622					
	M16c	16	PP766623					
	OM27	17	PP766624					
	OM35	17	PP766625		PP768685			
	OM59	18	PP766626					
	OM73	18	PP766627					
	OM122	19	PP766628					
	OM125	19	PP766629					
	OM132	20	PP766630					
	OM147	20	PP766631					
*Cladosporium* (Cladosporiaceae)								
*C. cladosporioides*	M4	27	PP766643	PP768710			PP768725	
*C. limoniforme*	OM52	28	PP766644		PP768690		PP768726	
*C. ramotenellum*	PT1a	29	PP766645		PP768691			
	PT4	29	PP766646	PP768711	PP768692		PP768727	
*Beauveria* (Cordycipitaceae)								
*B. pseudobassiana*	BF1	24	PP766639	PP768706				
*Neocucurbitaria* (Cucurbitariaceae)								
*N. keratinophila*	M4a	30	PP766647	PP768712				
*Candida* (Debaryomycetaceae)								
*C. boidinii*	Y1a	32	PP766649					
	Y2	33	PP766650					
	Y3	33	PP766651					
	Y8	34	PP766652					
	Y9	34	PP766653					
	Y5	35	PP766654					
	Y6	36	PP766655					
	Y7	37	PP766656					
	Y10	38	PP766657	PP768713				
	Y11	39	PP766658					
	Y12	40	PP766659					
	Y14	41	PP766660					
*Stagonosporopsis* (Didymellaceae)								
*S. ailanthicola*	OM34	31	PP766648					
*Geotrichum* (Dipodascaceae)								
*G. candidum*	OM5	43	PP766662					
	OM58	43	PP766663					
	OM62	43	PP766664					
	OM84	43	PP766665					
	GZ3	44	PP766666					
	G1	45	PP766667					
	G2	45	PP766668					
*Trichoderma* (Hypocreaceae)								
*T. amurcicola *sp. nov.	MF2a	23	PP766637	PP768704		PP768717	PP768723	PP768730
	MF2b	23	PP766638	PP768705		PP768718	PP768724	PP768731
*T. olivarum *sp. nov.	MF1a	22	PP766635	PP768702		PP768715	PP768721	PP768728
	MF1b	22	PP766636	PP768703		PP768716	PP768722	PP768729
*Pleurostoma* (Pleurostomataceae)								
*P. richardsiae*	M3	26	PP766641	PP768708	PP768688	PP768719		
	M3a	26	PP766642	PP768709	PP768689	PP768720		
*Barnettozyma* (Phaffomycetaceae)								
*B. californica*	Y4	42	PP766661	PP768714				
*Sarocladium* (Sarocladiaceae)								
*S. kiliense*	M10	25	PP766640	PP768707				
*Talaromyces* (Trichocomaceae)								
*T. nanjingensis*	M13	21	PP766632	PP768701	PP768686			
	Μ13a	21	PP766633		PP768687			
	Μ13c	21	PP766634					
BASIDIOMYCOTA								
*Fuscoporia* (Hymenochaetaceae)								
*F. ferrea*	M9a	48	PP766673					
*Pseudophlebia* (Meruliaceae)								
*P. setulosa*	OM106	46	PP766669					
	OM141	46	PP766670					
	PT3N	47	PP766671					
	PT5N	47	PP766672					
*Peniophora* (Peniophoraceae)								
*P. lycii*	Μ11	49	PP766676					
	M14	49	PP766677					
MUCOROMYCOTA								
*Mucor* (Mucoraceae)								
*M. circinelloides*	Z3	54	PP766678					
*M. pseudolusitanicus*	Z4	55	PP766679					
	Z6	55	PP766680					
	Z7	56	PP766681					
	Z9	50	PP766682					
*M. racemosus*	Z1	51	PP766683					
	Z5	51	PP766684					
	Z2	52	PP766685					
	Z8	53	PP766686					

**Table 2:** Enzyme indices (EIs) of selected fungal strains isolated from TPOMW on various substrates. Enzymatic activities were evaluated on cellulose-enriched agar (CEA), xylan-enriched agar (XEA), lignin-enriched agar (LEA), potato dextrose agar supplemented with guaiacol (PDA-G), and minimal salts agar supplemented with Remazol Brilliant Blue R (MS-RB), and were expressed in terms of semi-quantitative value categories ranging from no activity [depicted as ‘0’] to high activity [depicted as ‘3’]; value categories are placed at the base of this table. Columns indicate the fungal species identified, the number of strains tested vs. the number of strains isolated per species, the isolation conditions, e.g., substrate type [CEA, LEA, PDA, YPDA, with or without RBBR (-RB) or Econazole at 10 and 25 mg L^−1^ (-EC10 or -EC25)], inoculation method (SD: serial dilution; EC-SD, EL-SD, EY-SD: pre-enrichment with CEM, LEM, or YPDB followed by serial dilution; WU: modified Warcup plating), temperature (25 °C or 45 °C), and EI values on the five tested media. Strains with activity in all examined assays are presented in bold; species are presented in alphabetical order per phylum, then family, and finally genus (as in Table 1).

Species	Strain (Tested/Total)	Isolation Conditions	CEA	XEA	LEA	PDA-G	MS-RB
Ascomycota							
*Aspergillus filifer*	1/1	PDA/EC-SD/25	1	1	0	0	1
*Aspergillus fumigatus*	2/3	CEA, LEA/WU/45	1	1	0	0	0
*Aspergillus novoparasiticus*	2/2	PDA/EL-SD/25, 45	1–2	2	0	0	0–1
*Aspergillus sydowii*	1/1	CEA/SD/25	3	1	0	1	0
*Aspergillus tubingensis*	2/3	PDA, CEA/EC-SD, EL-SD, SD/25	2	1	0	0	1
*Aspergillus welwitschiae*	2/3	PDA-RB/EC-SD, EL-SD, SD/25, 45	0	1	0	0	1
*Aspergillus westerdijkiae*	1/1	PDA/EL-SD/25	1	2	0	0	1
*Penicillium crustosum*	5/9	CEA, CEA-EC25, LEA/WU, EL-SD, SD/25	1–3	1–2	0	0	0–1
*Penicillium kongii*	2/2	CEA/SD/25	2–3	1	0	0	1
*Penicillium paneum*	2/2	YPDA-EC25, LEA/WU/25	3	2	0	0	1
*Penicillium roqueforti*	9/14	LEA, CEA, PDA/SD, EL-SD, EY-SD, WU/25	1–3	1	0	0	0–2
*Cladosporium cladosporioides*	1/1	LEA/EL-SD/25	2	2	0	0	1
*Cladosporium limoniforme*	1/1	CEA/SD/25	**2**	**1**	**3**	**2**	**2**
*Cladosporium ramotenellum*	2/2	CEA/SD/25	**2–3**	**1–2**	**3**	**3**	**2**
*Beauveria pseudobassiana*	1/1	CEA/SD/25	3	3	0	3	3
*Neocucurbitaria keratinophila*	1/1	YPDA/SD/25	2	3	0	0	1
*Candida boidinii*	2/12	YPDA, YPDA-EC10, YPDA-EC25, CEA, CEA-EC10, LEA-EC25, PDA-EC25/EC-SD, EL-SD, EY-SD, WU/25	3	2–3	0	0	3
*Stagonosporopsis ailanthicola*	1/1	CEA/SD/25	0	0	0	0	0
*Geotrichum candidum*	6/7	CEA, YPDA, PDA-RB/SD, EC-SD, EL-SD/25	1–3	3	0	0	0–1
*Trichoderma amurcicola *sp. nov.	1/2	CEA/SD/25	1	1	0	1	1
*Trichoderma olivarum *sp. nov.	1/2	CEA/SD/25	1	1	0	0	0
*Pleurostoma richardsiae*	2/2	CEA, PDA/EC-SD/25	1–2	1–2	0	1	1–2
*Barnettozyma californica*	1/1	CEA/EL-SD/25	3	3	0	0	2
*Sarocladium kiliense*	1/1	CEA/EL-SD/25	1	2	0	0	1
*Talaromyces nanjingensis*	2/3	YPDA-EC10, YPDA-EC25/WU, EL-SD/25	1	1	0	0	0
Basidiomycota							
*Fuscoporia ferrea*	1/1	LEA/WU/25	**3**	**1**	**1**	**1**	**1**
*Pseudophlebia setulosa*	2/4	CEA/SD/25	**1–2**	**1**	**1–2**	**2–3**	**1–2**
*Peniophora lycii*	2/2	CEA-EC25/WU/25	1	3	2	0	2
Mucoromycota							
*Mucor circinelloides*	1/1	LEA/SD/25	1	2	0	0	1
*Mucor pseudolusitanicus*	4/4	PDA-RB, LEA, CEA/WU, EC-SD/25	1	1–2	0	0	1
*Mucor racemosus*	4/4	CEA, LEA, PDA/WU, EC-SD/25	1	0	0	0	0–1

EI value categories are as follows: on CEA, ‘1’ for ≤1.8, ‘2’ for 1.8–2.8, and ‘3’ for ≥2.8; on XEA, ‘1’ for ≤1.5, ‘2’ for 1.5–2.0, and ‘3’ for ≥2.0; on LEA, ‘1’ for ≤1.2, ‘2’ for 1.2–1.7, and ‘3’ for ≥1.7; on PDA-G, ‘1’ for ≤1.0, ‘2’ for 1.0–1.9, and ‘3’ for ≥1.9; and on PDA-RB, ‘1’ for ≤1.3, ‘2’ for 1.3–1.9, and ‘3’ for ≥1.9.

## Data Availability

The data presented in the manuscript are available on request from the corresponding author. In addition, sequences generated by this study are deposited in GenBank, and phylogenetic trees and pertinent data are deposited in TreeBASE at www.treebase.org, reference number 32208.

## Supplementary Materials

The following supporting information can be downloaded at https://www.mdpi.com/article/10.3390/jof11090687/s1: Figure S1. Phylogenetic tree for the genus *Mucor*, Mucoromycota (ITS; DS_MUC_, Supplementary Materials, Table S3), including strains recovered from TPOMW. Species identified are presented in colored boxes. Type strains are shown in bold black, while strains obtained in this study are marked in bold blue. Branch support values are shown where MLBS ≥ 65% and BPP ≥ 0.95; asterisks (*) denote MLBS = 100% and BPP = 1.00. Figure S2. Phylogenetic trees for fungal genera/species corresponding to strains (representing MOTUs) of Ascomycota recovered from TPOMW. The molecular markers and the respective datasets used appear in Table 1 and in Supplementary Materials, Table S3. Trees are presented in alphabetical order on the basis of the family name (Table 1): (**a**) *Aspergillus* (ITS and *tub*2; dataset DS_ASP_); (**b**) *Penicillium* (ITS and *tub*2; DS_PEN_); (**c**) *Cladosporium* (ITS and *act*; DS_CLA_); (**d**) *Candida boidinii* and *Barnettozyma californica* (ITS and *tef*1-α; DS_CA-BA_); (**e**) *Geotrichum* (ITS; DS_GEO_); (**f**) *Pleurostoma* (ITS, 28S, *tub*2, *tef*1-α and *rpb*2; DS_PLE_); (**g**) *Talaromyces* (ITS, *tub*2; DS_TAL_). Species identified are presented in colored boxes. Type strains are shown in bold black, while strains obtained in this study are marked in bold blue. Intrageneric sections are indicated along major branches. Branch support values are shown where MLBS ≥ 65% and BPP ≥ 0.95; asterisks (*) denote MLBS = 100% and BPP = 1.00. Table S1. Composition of solid and liquid media used for the isolation of fungi and enzymatic assays. For each medium, the respective concentrations (g L^−1^) are provided. Table S2. Primer pairs used for the amplification of fungal DNA markers. Includes targeted locus, primer names and sequences (5′–3′), expected amplicon size (bp), annealing temperature (°C), and corresponding references. Table S3. Characteristics of the sequence datasets used in the phylogenetic analyses of each fungal genus. Table S4. Reference sequences used in the phylogenetic analysis of the genus *Mucor*. For each strain, the sequence identifier, voucher code, and GenBank accession numbers. Superscripts next to voucher codes denote type status: H = holotype, T = type, I = isotype, EX-N = ex-neotype. Table S5. Reference sequences used in the phylogenetic analyses of strains in the genera *Aspergillus*, *Penicillium*, and *Talaromyces*. Listed for each strain are the sequence identifier, voucher code, and GenBank accession numbers for the examined loci. Superscripts next to voucher codes denote type status: H = holotype, T = type, N = neotype, E = epitype, S = syntype, EX = ex-type. Table S6. Reference sequences used in the phylogenetic analysis of the genus *Cladosporium*. For each strain, the sequence identifier, voucher code, and GenBank accession numbers for the examined loci. Superscripts next to voucher codes denote type status: H = holotype, N = neotype, E = epitype. Table S7. Reference sequences used in the phylogenetic analysis of the genera *Candida*, *Barnettozyma*, and *Geotrichum*. For each strain, the sequence identifier, voucher code, and GenBank accession numbers for the examined loci. Superscripts next to voucher codes denote type status: T = type, H = holotype. Table S8. Reference sequences used in the phylogenetic analysis of the genus *Pleurostoma*. For each strain, the sequence identifier, voucher code, and GenBank accession numbers for the examined loci. Superscripts next to voucher codes denote type status: H = holotype, T = type, I = isotype, and P = paratype. Table S9. Reference sequences used in the phylogenetic analysis of the genus *Trichoderma*. For each strain, the species name (as deposited in GenBank, if different from the accepted name), voucher code, and GenBank accession numbers for the examined loci. Superscripts next to voucher codes denote type status: H = holotype, T = type, E = epitype, EX = ex-type. Genome-derived sequences are marked with (G). Table S10. Enzymatic profiles of fungal strains isolated from TPOMW, with detailed results of enzyme indices (EI), laccase activity, and RBBR decolorization efficiency. Values represent means ± standard deviations based on three replicates. Strains with the highest enzymatic efficiency per category are highlighted in bold. “n.d.” denotes no detected enzymatic activity. References [91,92,93,94,95,96,97,98,99] are cited in the supplementary materials.
