# Supplementary material for: Diversity of Culturable Fungi in Two-Phase Olive Mill Waste, a Preliminary Evaluation of Their Enzymatic Potential, and Two New Trichoderma Species
Source: J Fungi (Basel). 2025 Sep 22;11(9):687. doi: 10.3390/jof11090687 (PMC12470466; doi:10.3390/jof11090687)
Supplement: Supplementary file 1 [file jof-11-00687-s001.zip › jof-3797647-supplementary.pdf]

Table S1. Composition of solid and liquid media used for isolation of fungi and enzymatic assays. For each medium, the respective concentrations (g L^{-1}) are provided [91].

	**Cellulose Enriched Medium (CEM)**	**Xylan Enriched Medium (XEM)**	**Lignin Enriched Medium (LEM)**	**Minimal Salts Medium (MSM)**	**Yeast Peptone Dextrose Broth (YPDB)**	**Synthetic Nutrient-Poor Broth (SNB)**
**carboxymethyl cellulose (CMC)**	2.0					
**birchwood xylan**		10.0				
**indulin AT***			1.0			
**$Ca(NO_3)_2{\cdot}4H_2O$**			0.05			
**$CaCl_2$**				0.025		
**K_2HPO_4**	1.0					1.0
**KH_2PO_4**			0.5	0.4		
**KCl**	0.5		0.1			0.5
**KNO_3**						0.5
**$MgSO_4$**	0.5		0.1	0.1		0.24
**Na_2HPO_4,**				1.6		
**$NaNO_3$**	2.0					
**NH_4NO_3**			0.1	0.5		
**$FeCl_3$**				0.0025		
**$FeSO_4{\cdot}7H_2O$**			0.05			
**dextrose**					20.0	0.2
**sucrose**						0.2
**peptone**	0.2	5.0			20.0	
**malt extract**			2.0			
**yeast extract**					10.0	
**pH (25°C)**	7.0 ± 0.2	5.5 ± 0.2	5.5 ± 0.2	7.2 ± 0.2	6.5 ± 0.2	5.5 ± 0.2
**solidified media**	**CEA**	**XEA**	**LEA**	**MSA**	**YPDA**	**SNA**
**agar**	18	18	18	18	18	18

* Indulin AT was disolved prior to sterilization in an alkaline solution (0.1M NaOH), was sterilized by filtration (0.22 μm), and was added aseptically in the rest of the medium after autoclaving, at a final concentration of 0.1% (w/v) (Martínez et al. 2005). The lignin solution consisted of 5 mL 1M KOH, 0.4 mL guaiacol, 1.0 g alkali lignin, and 10 mL dioxane (Merck & Co. Inc.).

Table S2. Primer pairs used for amplification of fungal DNA markers. Includes targeted locus, primer names and sequences (5′–3′), expected amplicon size (bp), annealing temperature (°C), and corresponding references.

Marker	Primer name	Primer sequence	Forward/ Reverse	Product length (bp)	Ta (°C)	Reference
**ITS**	**ITS1**	TCCGTAGGTGAACCTGCGG	F	600	52	[92]
	**ITS4**	TCCTCCGCTTATTGATATGC	R			[92]
**28S**	**LR0R**	ACCCGCTGAACTTAAGC	F	900	47	[93]
	**LR5**	TCCTGAGGGAAACTTCG	R			[93]
***tub*2**	**Btub2Fd**	GTBCACCTYCARACCGGYCARTG	F	550	52	[94]
	**Btub4Rd**	CCRGAYTGRCCRAARACRAAGTTGTC	R			[94]
***rpb*2**	**fRPB2-5F**	GAYGAYMGWGATCAYTTYGG	F	1000	55	[95]
	**fRPB2-7cR**	CCCATRGCTTGYTTRCCCAT	R			[95]
	**EF1-983F**	GCYCCYGGHCAYCGTGAYTTYAT	F	1000	58	[96]
***tef*1-a**	**EF1-2218R**	ATGACACCRACRGCRACRGTYTG	R			[96]
	**EF1-728F**	CATCGAGAAGTTCGAGAAGG	F	600	52	[97]
	**TEF1LLErev**	AACTTGCAGGCAATGTGG	R			[98]
cal	**CAL-228F**	GAGTTCAAGGAGGCCTTCTCCC	F	500	55	[97]
	**CAL-737R**	CATCTTTCTGGCCATCATGG	R			[97]
act	**TRI-ACT1**	TGGCACCACACCTTCTACAATGA	F	500	52	[99]
	**TRI-ACT2**	TCTCCTTCTGCATACGGTCGGA	R			[99]

Table S3. Characteristics of the sequence datasets used in the phylogenetic analyses of each fungal genus.

Dataset	DS_{MUC}	DS_{ASP}	DS_{PEN}	DS_{CLA}	DS_{CA-BA}
**Genera analyzed**	*Mucor*	*Aspergillus*	*Penicillium*	*Cladosporium*	*Candida – Barnettozyma*
**Molecular markers**	ITS	ITS, *tub2*	ITS, *tub2*	ITS, *act*	ITS, *tef1-α*
**Sequence no.**	19	36	24	15	13
**Length of alignment**	616	1240	1065	769	1691
**Constant characters**	543	828	887	667	1081
**Parsimony-informative characters**	58	388	151	83	406
**ML optimized Likelihood**	-1343.878	-5198.848	-2770.610	-2122.607	-6414.416
**Evolutionary model**	HKY+G+I	GTR+G+I	GTR+G	K2P+I	GTR+G+I
**BI no. of generations**	1040000	655000	555000	245000	400000
**BI 50% of calculated trees**	781	491	418	26	99
**Table reference sequence**	Table S4	Table S5	Table S5	Table S6	Table S7
**ML tree figure**	Figure S1	Figure S2a	Figure S2b	Figure S2c	Figure S2d

Dataset name	DS_{GEO}	DS_{PLE}	DS_{TAL}	DS_{TRI}
**Genera analyzed**	*Geotrichum*	*Pleurostoma*	*Talaromyces*	*Trichoderma*
**Molecular markers**	ITS	ITS, 28S, *rpb*2, *tef*1-*α*, *tub*2	ITS, *tub*2	ITS, *rpb*2, *tef*1-*α*, *cal*, *act*
**Sequence no.**	35	20	14	97
**Length of alignment**	445	4257	1023	4535
**Constant characters**	276	3271	952	3915
**Parsimony-informative characters**	122	535	40	475
**ML optimized Likelihood**	-2366.702	-13042.709	-2003.352	-14766.973
**Evolutionary model**	GTR+G+I	GTR+G+I	K2P+I	GTR+G+I
**BI no. of generations**	2010000	780000	1035000	48945000
**BI 50% of calculated trees**	1509	547	11	36711
**Table reference sequence**	Table S7	Table S8	Table S5	Table S9
**ML tree figure**	Figure S2e	Figure S2f	Figure S2g	Figure 5

Table S4. Reference sequences used in the phylogenetic analysis of the genus *Mucor*. For each strain, the sequence identifier, voucher code, and GenBank accession numbers. Superscripts next to voucher codes denote type status: H = holotype, T = type, I = isotype, EX-N = ex-neotype.

Species	Voucher code	ITS
***Mucor* (Mucoraceae)**		
M. atramentarius	CBS 202.28^{H}	JN205994
M. brunneogriseus	CBS 129.41^{T}	JF723573
M. circinelloides	CBS 195.68^{EX-N}	DQ118991
M. circinelloides	MZC-5	MN069563
M. circinelloides	CBS 121702	JN205966
M. ctenidius	CBS 293.66^{I}	JN205976
M. janssenii	CBS 205.68^{T}	HM999952
M. lusitanicus	CBS 108.17^{T}	JF439685
M. pseudolusitanicus	CBS 540.78^{H}	MF495059
M. racemosus f. *racemosus*	CBS 260.68^{T}	JN205898

Table S5. Reference sequences used in the phylogenetic analyses of strains in the genera *Aspergillus*, *Penicillium*, and *Talaromyces*. Listed for each strain are the sequence identifier, voucher code, and GenBank accession numbers for the examined loci. Superscripts next to voucher codes denote type status: H = holotype, T = type, N = neotype, E = epitype, S = syntype, EX = ex-type.

Species	Voucher code	ITS	TUB2
***Aspergillus* (Aspergillaceae)**			
**section *Circumdati***			
A. ochraceus	NRRL 398^{N}	AY373856	EF661322
A. ostianus	ATCC 16887^{N}	AY373858	
A. pallidofulvus	NRRL 4789^{H}	EF661423	EF661328
A. westerdijkiae	NRRL 3174^{H}	EF661427	EF661329
**section *Flavi***			
A. krugeri	PPRI 8986^{H}	MK450655	MK451098
A. mottae	CBS 130016^{H}	OL711742	MG517687
A. novoparasiticus	CBS 126849^{H}	OL711681	KY924673
A. sergii	CBS 130017^{H}	OL711674	MG517688
A. subflavus	CBS 143683^{H}	MH279429	MG517773
A. toxicarius	CBS 822.72^{T}	FJ491470	EF203163
**section *Fumigati***			
A. fischeri	NRRL 181^{N}	EF669936	EF669796
A. fumigatus	NRRL 163^{T}	EF669931	LC589344
A. fumigatus var. *ellipticus*	ATCC 16903^{T}	KC689316	HQ588168
A. oerlinghausenensis	CBS 139183^{H}	KT359601	KT359603
**section *Nigri***			
A. costaricensis	CBS 115574^{H}	FJ629326	AY820014
A. foetidus	CBS 121.28^{N}	MH854949	FJ491690
A. luchuensis	KACC 46772^{T}	JX500081	JX500062
A. neoniger	NRRL 62634^{T}	KC796401	KC796361
A. niger	ATCC 16888^{N}	FJ195350	KU897009
A. piperis	CBS 112811^{H}	FJ629352	
A. tubingensis	NRRL 4875^{T}	EF661193	EF661086
A. welwitschiae	CBS 139.54^{EX-E}	FJ629340	MN969369
**section *Nidulantes***			
A. astellatus	NRRL 2396^{S}	EF652446	EF652270
A. chinensis	IFM 54282^{H}	AB249003	AB248345
A. filifer	CBS 113636^{H}	EU448277	EF428372
A. griseoaurantiacus	CBS 138191^{H}	KJ775553	KJ775086
A. stellatus	NRRL 1858^{E}	EF652426	EF652250
A. sydowii	CBS 593.65^{N}	AB267812	EF428373
A. versicolor	CBS 583.65^{N}	EU076360	XM_040811307
***Penicillium* (Aspergillaceae)**			
**section *Brevicompacta***			
P. brevicompactum	NRRL 2011^{N}	AY484912	DQ645784
P. brevicompactum	ATHUM 5048	FJ004277	FJ004387
P. kongii	AS3.15329^{H}	KC427191	KC427171
P. neocrassum	NRRL 35639^{H}	DQ645805	DQ645794
P. patris-mei	CBS 210.28^{EX-T}	KC411694	
**section *Fasciculata***			
P. commune	CBS 311.48^{I}	AY213672	MN969377
P. crustosum	FRR 1669^{N}	AY373907	MN969379
P. fuscoglaucum	CBS 261.29^{I}	MH855062	FJ930977
P. solitum	CBS 424.89^{T}		MN969398
P. solitum	CBS 14786	HQ225713	AY674355
**section *Roquefortorum***			
P. carneum	CBS 112297^{T}	HQ442338	AY674386
P. mediterraneum	FMR 15188^{H}	LT899784	LT898291
P. paneum	CBS 101032^{T}	HQ442346	AY674387
P. psychrosexuale	CBS 128137^{H}	HQ442345	HQ442356
P. roqueforti	CBS 221.30^{N}	HQ442347	MN969396
***Talaromyces* (Trichocomaceae)**			
T. brevis	CBS 141833^{H}	MN864269	MN863338
T. californicus	NRRL 58168^{H}	MH793056	MH792928

T. californicus	NRRL 58661	MH793060	MH792932
T. liani	CBS 225.66[T]	MH858781	JX091380
T. liani	NRRL 3380[T]	MH793037	MH792909
T. louisianensis	NRRL 35823[H]	MH793052	MH792924
T. malicola	NRRL 3724[H]	MH909513	MH909406
T. muroii	CBS 756.96[H]	MN431394	KJ865727
T. nanjingensis	JP-NJ4[H]	MW130720	MW147759
T. pinophilus	CBS 631.66[N]	JN899382	JX091381
T. pinophilus	Y-94	AB474749	AB773823
T. veerkampii	CBS 500.78[H]	KF741984	KF741918

Table S6. Reference sequences used in the phylogenetic analysis of the genus *Cladosporium*. For each strain, the sequence identifier, voucher code, GenBank accession numbers for the examined loci. Superscripts next to voucher codes denote type status: H = holotype, N = neotype, E = epitype.

Species	Voucher code	ITS	ACT
***Cladosporium* (Cladosporiaceae)**			
***C. cladosporioides* complex**			
C. austroafricanum	CBS 140481[H]	KT600381	KT600577
C. cladosporioides	CBS 112388[N]	HM148003	HM148490
C. needhamense	CPC 22353[H]	MF473142	MF473991
C. phaenocomae	CBS 128769[H]	JF499837	JF499881
C. xylophilum	CBS 125997[H]	HM148230	HM148721
***C. herbarum* complex**			
C. colombiae	CBS 274.80B[H]	FJ936159	FJ936166
C. fildesense	F09-T12-1[H]	JX845290	MN233632
C. limoniforme	CBS 140484[H]	KT600397	KT600592
C. prolongatum	CGMCC3.18036[H]	KX938394	KX938377
C. ramotenellum	CBS 121628c[H]	EF679384	EF679538
C. tenellum	CBS 121634[H]	EF679401	EF679555
C. variabile	CBS 121636[E]	EF679402	EF679556

Table S7. Reference sequences used in the phylogenetic analysis of the genera *Candida*, *Barnettozyma*, and *Geotrichum*. For each strain, the sequence identifier, voucher code, GenBank accession numbers for the examined loci. Superscripts next to voucher codes denote type status: T = type, H = holotype.

Species	Voucher code	ITS	TEF1-α
***Candida* (Debaryomycetaceae)**			
B. californica	CBS 252[T]	DQ137886	EF552500
B. hawaiiensis	CBS 8760[H]	KY101728	EF552502
B. xylosiphila	NBRC 110202[H]	LC012021	LC012023
***Barnettozyma* (Phaffomycetaceae)**			
C. colombiae	CBS 274.80B[H]	FJ936159	FJ936166
C. fildesense	F09-T12-1[H]	JX845290	MN233632
C. limoniforme	CBS 140484[H]	KT600397	KT600592
C. prolongatum	CGMCC3.18036[H]	KX938394	KX938377
C. ramotenellum	CBS 121628c[H]	EF679384	EF679538
C. tenellum	CBS 121634[H]	EF679401	EF679555
C. variabile	CBS 121636[E]	EF679402	EF679556
***Geotrichum* (Dipodascaceae)**			
G. aggregatus	CBS 175.53[T]	OP765499	
G. candidum	CBS 615.84[T]	OP765493	
G. candidum	CBS 178.71[T]	KF984491	
G. candidum	CBS 180.33	JN974289	
G. candidum	LMA-48	JF262188	
G. candidum	TOM_YEAST	KF112070	
G. candidum	CBS 11620	KY103455	
G. candidum	CBS 11628	KY103456	
G. candidum	361-1	OP758542	
G. candidum	48h1-4	OP758543	
G. candidum (as *D. australiensis*)	V/2/08	HQ115737	
G. candidum (as *Geotrichum* sp.)	TU-GM11	DQ325460	
G. candidum (as *G. bryndzae*)	CBS 11176[T]	JN974290	
G. candidum (as *G. bryndzae*)	PMM09-440L	KP132251	
G. candidum (as *G. silvicola*)	CBS 9194[T]	HE663405	
G. carabidarus	CBS 9891[T]	KY103462	
G. citri-aurantii	CBS 176.89[T]	OP821147	
G. cucujoidarus	ATCC MYA-4341[T]	FJ196774	
G. europaeum	CBS 866.68[T]	OP765498	
G. fermentans	CBS 439.83[T]	OP765500	
G. galactomycetum	CBS 772.71[T]	OP765494	
G. galactomycetum	CBS 774.71	JN974293	
G. galactomycetum	DAOM 216615	JN942847	
G. ghanensis	CBS 11010[H]	KY103463	
G. histeridarus	ATCC MYA-4340[T]	FJ196773	
G. macrosporus	CBS 259.82[T]	OP765501	
G. phurueaensis	CBS 11418[T]	OP765496	
G. restrictus	CBS 111234[H]	EF126738	

Table S8. Reference sequences used in the phylogenetic analysis of the genus *Pleurostoma*. For each strain, the sequence identifier, voucher code, GenBank accession numbers for the examined loci. Superscripts next to voucher codes denote type status: H = holotype, T = type, I = isotype, P = paratype.

Species	Voucher code	ITS	28S	TUB2	RPB2	TEF1-α
Pleurostoma hongkongense	HKU 44[H]	MT153150		MT501300		
P. ochraceum	CBS 131321[H]	MT153175	MT158397	MT501326		
P. ootheca	CBS 115329[I]/ AFTOL-ID 5085	MT153174	MT158396	MT501325	HQ878606	FJ238420
P. ootheca	KUN-HKAS 122679	OM017217	OM017219	ON468660		
P. repens	CBS 294.39[I]	MT153176		MT501327		
P. richardsiae	CBS 270.33[I]	MT153151	MT158373	MT501301	HQ878607	
P. richardsiae	CBS 295.39	MH856019	MH867520			
P. richardsiae	CBS 310.49	MH856539	MH868063			
P. richardsiae	CBS 842.69	MH859454	MH871232			
P. richardsiae	KARE488	MT645621		MT734998		
P. richardsiae	KARE518	MT645622		MT734999		
P. richardsiae	KARE1868	MT645645		MT735022		
P. richardsiae	P2BA	OP028963				
P. richardsiae (as *Phialophora calyciformis*)	CBS 302.62[T]/ A177	AY249077	AY249090			
Calosphaeria pulchella	CBS 115999[I]/ AFTOL-ID 5086	EU367451	AY761075	KT716476	GU180661	FJ238421
Flabellascus tenuirostris	CBS 138680[H]	KT716466	KT716457	KT716488	KT716475	
Jattaea ribicola	CBS 139779[H]	KT716463	KT716454	KT716480		
J. algeriensis	CBS 120871[T]/ STE-U 6201	EU367446		EU367466	HQ878603	

Table S9. Reference sequences used in the phylogenetic analysis of the genus *Trichoderma*. For each strain, the species name (as deposited in GenBank, if different from the accepted name), voucher code, GenBank accession numbers for the examined loci. Superscripts next to voucher codes denote type status: H = holotype, T = type, E = epitype, EX = ex-type. Genome-derived sequences are marked with (G).

Taxa	Voucher code	ITS	RPB2	TEF1a	CAL	ACT
***Trichoderma* (Hypocreaceae)**						
T. afarasin	CBS 130755^{EX}/ GJS 99-227/ IMI 393967	AY027784		AF348093	FJ442388	
T. afarasin	CBS 130742/ DIS 314F	FJ442259	FJ442778	FJ463400	FJ442312	
T. afroharzianum	CBS 124620^{EX}/ GJS 04-186	FJ442265	FJ442691	FJ463301	FJ442370	
T. afroharzianum	IIPRTh-33 (G)	PRJNA770175	PRJNA770175	PRJNA770175		PRJNA770175
T. aggressivum	DAOM 222156^{H}	AF443911	FJ442752		AF442860	FJ442438
T. aggressivum	CBS 689.94	FJ442606	FJ442706	FJ467645	FJ442280	FJ442437
T. amurcicola (as *T. guizhouense*)	S278		KF134791	KF134799		
T. anaharzianum	YMF1.00383^{H}	MH113931	MH158995	MH183182		
T. asiaticum	YMF 1.00352^{H}/ CGMCC 3.19085	MH113930	MH158994	MH183183		
T. asiaticum	YMF 1.00168	MH262582	MH262575	MH236492		
T. atrobrunneum	CBS 548.92^{H}/ GJS 92-110	AF443924		AF443942		AF442850
T. atrobrunneum	GJS 04-67	FJ442273	FJ442724	FJ463360	FJ442329	
T. auriculariae	JZBQT1Z7^{H}	ON653396	ON649949	ON649896		
T. auriculariae	JZBQT1Z8	ON653397	ON649950	ON649897		
T. austroindianum	BAFC 3583^{H}/ VAB-T050			MH352421	MH337370	MH337371
T. austroindianum (as *T. camerunense*)	BAFC 3844			MG822709	MG822712	MG822715
T. azevedoi	CEN1422^{H}	MK714902	MK696821	MK696660	MK696714	
T. azevedoi	CEN1423	MK714903	MK696822	MK696661	MK696715	MK696769
T. bannaense	HMAS 248840^{H}/ TC564	KY687923	KY687979	KY688037		
T. bannaense	HMAS 248865/ TC943	KY687948	KY688003	KY688038		
T. botryosum	COAD 2422^{H}		MK044212	MK044119		
T. botryosum	COAD 2401		MK044181	MK044088		
T. breve	HMAS 248844^{H}	KY687927	KY687983	KY688045		
T. breve (as *T. brevicrassum*)	TC967^{T} (G)		PRJNA685005	PRJNA685005	PRJNA685005	PRJNA685005
T. caespitosum	JZBQT1Z6^{T}		OP832383	OP832398		
T. caespitosum	JZBQT1Z12		OP832384	OP832399		
T. camerunense	CBS 138272^{EX}/GJS 99-230	AY027780		AF348107	AF442875	AF442842
T. camerunense	GJS 99-231	AY027783		AF348108	AF442874	AF442841
T. cf. *atrobrunneum* (as *T. atrobrunneum*)	ITEM 908 (G)	PRJNA428936	PRJNA428936	PRJNA428936	PRJNA428936	PRJNA428936
T. cf. *atrobrunneum* (as *T. lixii*)	C.P.K. 1934	EF392746	FJ179608	FJ179573		
T. cf. *guizhouense* #1 (as *T. guizhouense*)	GJS 97-28^{EX}/ NBRC 30608/ IFO 30608	DQ018116		AY937440	FJ442379	FJ442532
T. cf. *guizhouense* #1 (as *T. guizhouense*)	BAFC 4356			MG797485	MG797490	MG797495
T. cf. *guizhouense* #2 (as *T. guizhouense*)	NJAU 4742	PRJNA314460	PRJNA314460	PRJNA314460	PRJNA314460	PRJNA314460
T. cf. *guizhouense* #2 (as *T. guizhouense*)	BAFC 4370			MG797486	MG797491	MG797496
T. cf. *harzianum* (as *T. harzianum*)	CBS 226.95^{N} (G)	PRJNA207867	PRJNA207867	PRJNA207867	PRJNA207867	PRJNA207867
T. cf. *harzianum* (as *T. harzianum*)	CGMCC 207.39		MZ603731	MZ603732		
T. cf. *simmonsii* (*T. simmonsii*)	GH-Sj1 (G)	PRJNA645793	PRJNA645793	PRJNA645793	PRJNA645793	PRJNA645793

T. cornaroae	MST F22317 ^{H}	PP476954	PP475471	PP475472		
T. endophyticum (as *T. harzianum*)	CBS 130729^{H}/ DIS 217A/ IMI 395208	FJ442243		FJ463319	FJ442292	
T. endophyticum (as *T. harzianum*)	DIS 217H	FJ442242	FJ442721	FJ463314	FJ442293	FJ442446
T. guizhouense	HGUP0038^{H}/ CBS 131803	JN191311	JQ901400	JN215484		
T. guizhouense (as *T. harzianum*)	GJS 06-133	FJ442636		FJ463337	FJ442349	FJ442566
T. harzianum	CBS 226.95^{N}	AY605713	AF545549	AF348101		
T. harzianum (as *T. lixii*)	MUT 3171	PRJNA514353	PRJNA514353		PRJNA514353	PRJNA514353
T. hortense	GJS 08-116^{H}/ BAFC4291			MH253895	MH253896	MH253897
T. inhamatum	CBS 273.78^{EX}/ GJS 95-39/ IMI 287526	FJ442680	FJ442725	AF348099	FJ577683	
T. jaklitschii	CP61-2^{T}		MW480149	MW480140		MW480165
T. jaklitschii	CP62-2		MW480151	MW480141		MW480168
T. lentiforme	CBS 100542$^{EX\text{-}E}$/ GJS 98-6/ IMI 393968	AF469189		AF469195	AF469191	AF469193
T. lentiforme	Dis 110a^{EX}/ CBS 130726	FJ442681	FJ442786	FJ851872	FJ442287	FJ442440
T. lentinulae	HMAS 248256^{H}/ CGMCC 3.19847	MN594469	MN605867	MN605878		
T. lentinulae	CGMCC 3.19848	MN594470	MN605868	MN605879		
T. lixii	CBS 110080^{E}/ GJS 97-96/ ATCC MYA-2478/ BPI 745654	AF443920	KJ665290	FJ716622	FJ577772	AF442839
T. macrochlamydosporum	JZBQT5Z1^{T}	ON653399	ON649955	ON649902		
T. macrochlamydosporum	JZBQT6Z1	ON653400	ON649957	ON649904		
T. neotropicale	CBS 130633^{H}/ GJS 11-185/ LA11	HQ022407		HQ022771	KP115279	
T. neotropicale (as *T. lentiforme*)	CFAM-422 (G)		PRJNA473534	PRJNA473534	PRJNA473534	PRJNA473534
T. notatum	JZBQT1Z5^{T}		OP832381	OP832396		
T. notatum	JZBQT1Z11		OP832397	OP832382		
T. olivarum (as *T. harzianum*)	GJS 04-227	FJ442266		FJ463383	FJ442368	FJ442491
T. peruvianum	CP15-9		MW480154	MW480146		MW480170
T. peruvianum	CP15-2		MW480153	MW480145		MW480169
T. pholiotae	JZBQH12^{H}		ON649972	ON649919		
T. pholiotae	JZBQH13		ON649973	ON649920		
T. pingquanense	JZBQT7Z10^{T}	ON65340	ON649961	ON649908		
T. pingquanense	JZBQT7Z11		ON649962	ON649909		
T. pollinicola	LC11682^{T}	MF939592	MF939604	MF939619	MF939586	
T. pollinicola (as *T. harzianum*)	TRA2-5	MW325798	MW331878	MW267316		
T. pseudoasiaticum	YMF 1.06200^{H}/ YMF 1.6178/ CCTCC AF 2021061	MN977792	MT052183	MT070155		
T. pseudopyramidale	COAD 2426		MK044224	MK044131	MK084870	
T. pseudopyramidale	COAD 2433		MK044250	MK044157	MK084869	
T. pyramidale	CBS 135574$^{EX/H}$/ S73		KJ665334	KJ665699		
T. pyramidale	S119			KJ665696		
T. rifaii	CBS 130746^{EX}/ DIS 355B	FJ442663		FJ463324		
T. rugulosum	SFC20180301-001^{H}	MH050353	MH025986	MH025984		
T. rugulosum (as *T. semiorbis*)	FJ059 (G)	PRJNA756961	PRJNA756961	PRJNA756961		PRJNA756961
T. simile	YMF 1.06201^{H}	MN977793	MT052184	MT070154		
T. simile	YMF 1.6180	MN977794	MT052185	MT070153		
T. simmonsii	CBS 130431^{EX}/ GJS 91-138	AF443917	FJ442757	AF443935	AF442869	AF442836
T. simmonsii	S7		KJ665337	KJ665719		
T. subvermifimicola	JZBQT4Z1^{T}	ON653398	ON649952	ON649899		

T. subvermifimicola	JZBQT4Z2		ON649953	ON649900		
T. syagri (*T. camerunense*)	BAFC 4357 [H]			MG822711	MG822714	MG822717
T. syagri (*T. camerunense*)	BAFC 4371			MG822710	MG822713	MG822716
T. tongzhouense	JZBQT1Z1[T]	ON653394	ON649945	ON649892		
T. tongzhouense	JZBQT1Z4	ON653395	ON649948	ON649895		
T. vermifimicola	HMAS 248255[H]	MN594473	MN605871	MN605882		
T. vermifimicola	CGMCC 3.19850	MN594472	MN605870	MN605881		
T. xixiacum	HMAS 248253[H]/ CGMCC 3.19697	MN594476	MN605874	MN605885		
T. xixiacum	CGMCC 3.19698	MN594477	MN605875	MN605886		
T. zelobreve	HMAS 248254[H]/ CGMCC 3.19695	MN594474	MN605872	MN605883		
T. zelobreve	CGMCC 3.19696	MN594475	MN605873	MN605884		
T. zeloharzianum	YMF1.00268[T]	MH113932	MH158996	MH183181		

Table S10. Expanded enzymatic profiles of fungal strains isolated from TPOMW, with detailed results of enzyme indices (EI), laccase activity, and RBBR decolorization efficiency. Values represent means ± standard deviations based on three replicates. Strains with the highest enzymatic efficiency per category are highlighted in bold. "n.d." denotes no detected enzymatic activity.

Species	Strain	CEA	XEA	LEA	PDA-G	MS-RB
**ASCOMYCOTA**						
Aspergillus filifer	**LGAM SOW_A5a**	1.03±0.15	1.20±0.12	n.d.	n.d.	1.15±0.06
Aspergillus fumigatus	**LGAM SOW_A1**	1.10±0.14	1.24±0.12	n.d.	n.d.	n.d.
	**LGAM SOW_A3**	1.08±0.11	1.15±0.09	n.d.	n.d.	n.d.
Aspergillus novoparasiticus	**LGAM SOW_T1**	2.33±0.33	1.64±0.23	n.d.	n.d.	1.10±0.03
	**LGAM SOW_T2**	1.16±0.21	1.62±0.30	n.d.	n.d.	n.d.
Aspergillus sydowii	**LGAM SOW_PT1b**	**4.50±0.23**	1.33±0.24	n.d.	0.55±0.09	n.d.
Aspergillus tubingensis	**LGAM SOW_A6**	2.55±0.18	1.17±0.08	n.d.	n.d.	1.10±0.02
	**LGAM SOW_A7**	2.70±0.16	1.20±0.12	n.d.	n.d.	1.12±0.04
Aspergillus welwitschiae	**LGAM SOW_A4**	n.d.	1.18±0.05	n.d.	n.d.	1.10±0.04
	**LGAM SOW_A8**	n.d.	1.25±0.11	n.d.	n.d.	1.12±0.03
Aspergillus westerdijkiae	**LGAM SOW_A9a**	1.03±0.12	1.64±0.14	n.d.	n.d.	1.13±0.03
Penicillium crustosum	**LGAM SOW_M1a**	1.77±0.24	1.69±0.33	n.d.	n.d.	1.05±0.01
	**LGAM SOW_M2**	2.90±0.23	1.82±0.28	n.d.	n.d.	1.10±0.03
	**LGAM SOW_M6**	2.00±0.28	1.71±0.21	n.d.	n.d.	1.05±0.02
	**LGAM SOW_P1**	2.14±0.26	1.38±0.14	n.d.	n.d.	1.05±0.03
	**LGAM SOW_P5**	**3.82±0.22**	1.91±0.36	n.d.	n.d.	n.d.
Penicillium kongii	**LGAM SOW_PT2**	**2.84±0.16**	1.10±0.03	n.d.	n.d.	1.20±0.08
	**LGAM SOW_PT5**	1.88±0.13	1.10±0.08	n.d.	n.d.	1.21±0.08
Penicillium paneum	**LGAM SOW_M8**	**3.13±0.20**	1.70±0.22	n.d.	n.d.	1.19±0.04
	**LGAM SOW_M15**	**4.29±0.17**	1.88±0.31	n.d.	n.d.	1.17±0.07
Penicillium roqueforti	**LGAM SOW_M5**	1.10±0.21	1.22±0.12	n.d.	n.d.	n.d.
	**LGAM SOW_M12**	3.00±0.25	1.33±0.13	n.d.	n.d.	n.d.
	**LGAM SOW_M16c**	1.31±0.14	n.d.	n.d.	n.d.	n.d.
	**LGAM SOW_OM35**	1.50±0.20	n.d.	n.d.	n.d.	1.30±0.08
	**LGAM SOW_OM59**	2.00±0.25	n.d.	n.d.	n.d.	1.30±0.03
	**LGAM SOW_OM73**	1.08±0.28	n.d.	n.d.	n.d.	1.30±0.05
	**LGAM SOW_OM125**	1.70±0.27	n.d.	n.d.	n.d.	1.27±0.08
	**LGAM SOW_OM132**	1.60±0.28	n.d.	n.d.	n.d.	1.40±0.06
	**LGAM SOW_OM147**	1.25±0.19	n.d.	n.d.	n.d.	1.33±0.05
Talaromyces nanjingensis	**LGAM SOW_M13a**	1.22±0.08	1.14±0.08	n.d.	n.d.	n.d.
	**LGAM SOW_M13c**	1.17±0.18	1.16±0.04	n.d.	n.d.	n.d.
Trichoderma olivarum	**LGAM SOW_MF1a**	1.57±0.17	1.45±0.13	n.d.	n.d.	n.d.
Trichoderma amurcicola	**LGAM SOW_MF2a**	1.74±0.06	1.49±0.19	n.d.	0.71±0.10	1.10±0.04
Beauveria pseudobassiana	**LGAM SOW_BF1**	**4.00±0.28**	**3.50±0.22**	n.d.	**2.67±0.03**	**2.50±0.22**
Sarocladium kiliense	**LGAM SOW_M10**	1.43±0.02	1.82±0.25	n.d.	n.d.	1.07±0.03
Pleurostoma richardsiae	**LGAM SOW_M3**	1.83±0.03	1.43±0.10	n.d.	1.00±0.02	1.38±0.09
	**LGAM SOW_M3a**	1.63±0.04	1.60±0.15	n.d.	0.90±0.03	1.20±0.04
Cladosporium cladosporioides	**LGAM SOW_M4**	2.67±0.15	1.60±0.24	n.d.	n.d.	1.11±0.03
Cladosporium limoniforme	**LGAM SOW_OM52**	2.55±0.18	1.50±0.14	**1.70±0.04**	1.80±0.13	1.50±0.05
Cladosporium ramotenellum	**LGAM SOW_PT1a**	2.71±0.13	1.21±0.06	**1.80±0.12**	**3.17±0.05**	1.50±0.07
	**LGAM SOW_PT4**	3.13±0.21	1.67±0.13	**2.00±0.07**	**2.00±0.08**	1.50±0.03
Neocucurbitaria keratinophila	**LGAM SOW_M4a**	2.08±0.09	**2.33±0.27**	n.d.	n.d.	1.05±0.03
Stagonosporopsis ailanthicola	**LGAM SOW_OM34**	n.d.	n.d.	n.d.	n.d.	n.d.
Candida boidinii	**LGAM SOW_Y5**	3.00±0.05	1.67±0.21	n.d.	n.d.	1.67±0.09
	**LGAM SOW_Y10**	3.00±0.02	2.00±0.30	n.d.	n.d.	**2.00±0.05**
Barnettozyma californica	**LGAM SOW_Υ4**	3.00±0.03	2.00±0.13	n.d.	n.d.	1.50±0.02
Geotrichum candidum	**LGAM SOW_OM5**	1.10±0.20	**2.35±0.15**	n.d.	n.d.	n.d.
	**LGAM SOW_OM62**	1.15±0.08	2.20±0.18	n.d.	n.d.	n.d.
	**LGAM SOW_OM84**	1.10±0.15	2.25±0.29	n.d.	n.d.	n.d.
	**LGAM SOW_GZ3**	1.80±0.04	**2.37±0.13**	n.d.	n.d.	1.10±0.03
	**LGAM SOW_G1**	1.90±0.08	**3.00±0.17**	n.d.	n.d.	n.d.
	**LGAM SOW_G2**	2.91±0.17	**2.65±0.12**	n.d.	n.d.	n.d.
**BASIDIOMYCOTA**						
Pseudophlebia setulosa	**LGAM SOW_PT3N**	1.04±0.04	1.30±0.14	1.25±0.08	1.50±0.02	0.63±0.04
	**LGAM SOW_PT5N**	1.88±0.23	1.20±0.08	1.20±0.03	**3.00±0.08**	1.50±0.08
Fuscoporia ferrea	**LGAM SOW_M9a**	**4.44±0.21**	1.09±0.03	1.20±0.06	0.81±0.15	0.30±0.05
Peniophora lycii	**LGAM SOW_M11**	1.50±0.13	**2.38±0.29**	1.25±0.13	n.d.	1.43±0.07
	**LGAM SOW_M14**	1.50±0.31	**2.67±0.14**	1.22±0.03	n.d.	1.40±0.04
**MUCOROMYCOTA**						
Mucor circinelloides	**LGAM SOW_Z3**	1.05±0.02	1.70±0.22	n.d.	n.d.	1.06±0.03
	**LGAM SOW_Z4**	1.06±0.01	1.41±0.13	n.d.	n.d.	1.15±0.05

Mucor pseudolusitanicus	**LGAM SOW_Z6**	1.11±0.08	1.60±0.19	n.d.	n.d.	1.13±0.02
	**LGAM SOW_Z7**	1.10±0.02	1.50±0.09	n.d.	n.d.	1.11±0.02
	**LGAM SOW_Z9**	1.15±0.05	1.75±0.23	n.d.	n.d.	1.10±0.04
Mucor racemosus	**LGAM SOW_Z1**	1.05±0.24	n.d.	n.d.	n.d.	n.d.
	**LGAM SOW_Z2**	1.21±0.38	n.d.	n.d.	n.d.	n.d.
	**LGAM SOW_Z5**	0.98±0.22	n.d.	n.d.	n.d.	n.d.
	**LGAM SOW_Z8**	1.20±0.17	n.d.	n.d.	n.d.	1.10±0.03

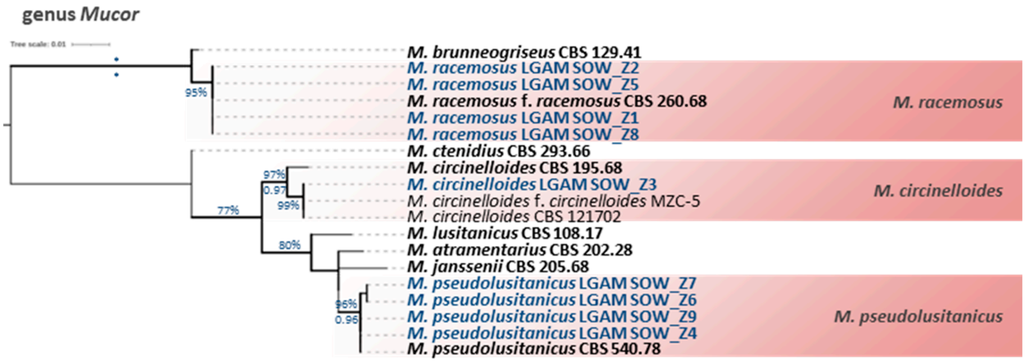

Figure S1. Phylogenetic tree for the genus *Mucor*, Mucoromycota (ITS; DS_{MUC}, Supplementary Material, Table S3), including strains recovered from TPOMW. Species identified are presented in colored boxes. Type strains are shown in bold black, while strains obtained in this study are marked in bold blue. Branch support values are shown where MLBS ≥ 65% and BPP ≥ 0.95; asterisks (*) denote MLBS = 100% and BPP = 1.00.

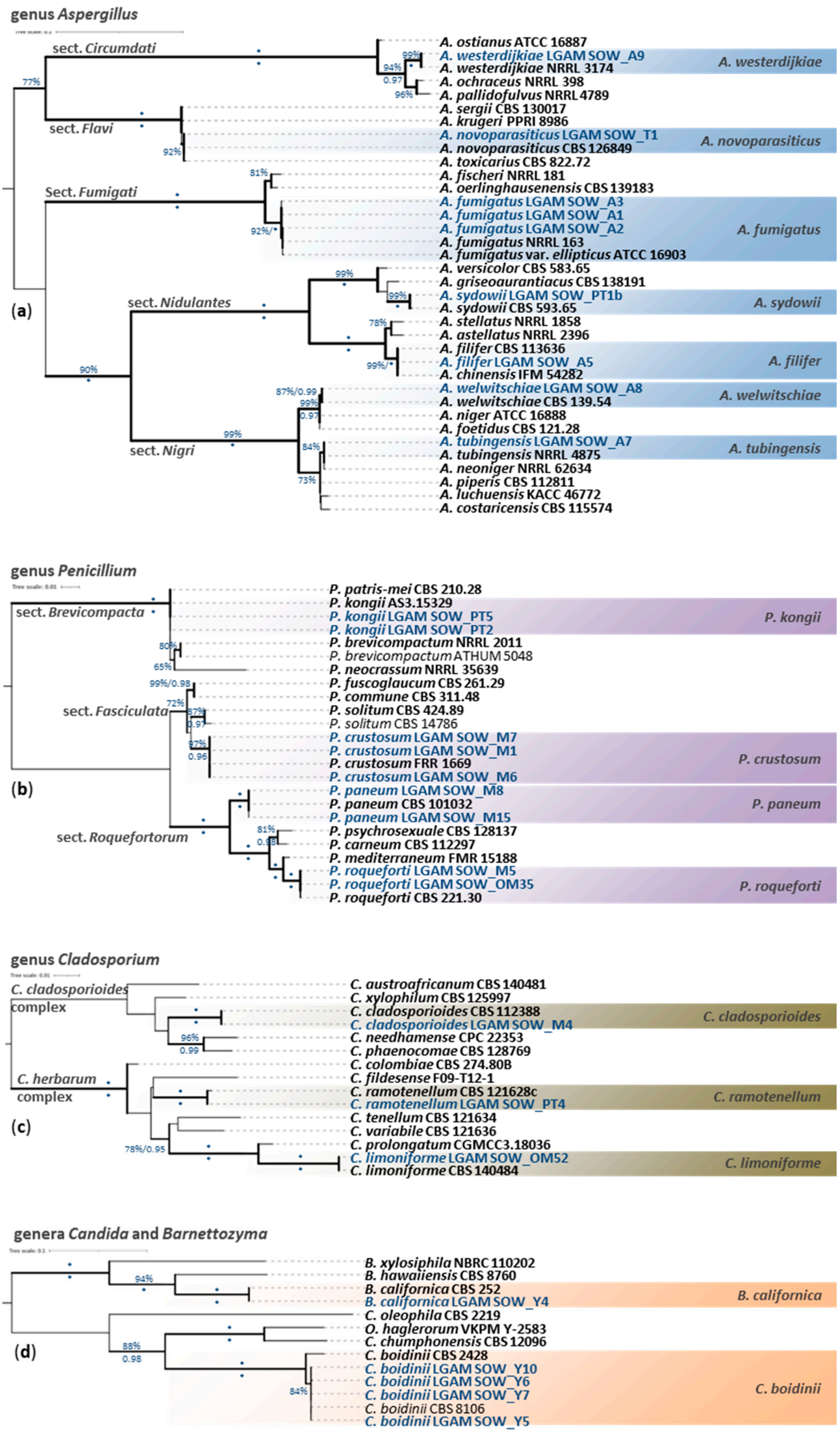
genus Aspergillus
sect. Circumdati
A. ostianus ATCC 16887
A. westerdijkiae LGAM SOW_A9
A. westerdijkiae NRRL 3174
A. ochraceus NRRL 398
A. pallidofulvus NRRL 4789
A. westerdijkiae
98%
94%
0.97
96%
77%
sect. Flavi
A. sergii CBS 130017
A. krugeri PPRI 8986
A. novoparasiticus LGAM SOW_T1
A. novoparasiticus CBS 126849
A. toxicarius CBS 822.72
A. novoparasiticus
92%
Sect. Fumigati
81%
A. fischeri NRRL 181
A. oerlinghausenensis CBS 139183
A. fumigatus LGAM SOW_A3
A. fumigatus LGAM SOW_A1
A. fumigatus LGAM SOW_A2
A. fumigatus NRRL 163
A. fumigatus var. ellipticus ATCC 16903
A. fumigatus
92%/*
(a)
sect. Nidulantes
99%
A. versicolor CBS 583.65
A. griseoaurantiacus CBS 138191
A. sydowii LGAM SOW_PT1b
A. sydowii CBS 593.65
A. sydowii
99%
78%
A. stellatus NRRL 1858
A. astellatus NRRL 2396
A. filifer CBS 113636
A. filifer LGAM SOW_A5
A. chinensis IFM 54282
A. filifer
99%/*
90%
87%/0.99
A. welwitschiae LGAM SOW_A8
A. welwitschiae CBS 139.54
A. welwitschiae
99%
0.97
A. niger ATCC 16888
A. foetidus CBS 121.28
sect. Nigri
99%
84%
A. tubingensis LGAM SOW_A7
A. tubingensis NRRL 4875
A. tubingensis
A. neoniger NRRL 62634
73%
A. piperis CBS 112811
A. luchuensis KACC 46772
A. costaricensis CBS 115574
genus Penicillium
sect. Brevicompacta
P. patris-mei CBS 210.28
P. kongii AS3.15329
P. kongii LGAM SOW_PT5
P. kongii LGAM SOW_PT2
P. kongii
80%
65%
P. brevicompactum NRRL 2011
P. brevicompactum ATHUM 5048
P. neocrassum NRRL 35639
99%/0.98
P. fuscoglaucum CBS 261.29
72%
P. commune CBS 311.48
sect. Fasciculata
87%
0.97
P. solitum CBS 424.89
P. solitum CBS 14786
97%
0.96
P. crustosum LGAM SOW_M7
P. crustosum LGAM SOW_M1
P. crustosum FRR 1669
P. crustosum LGAM SOW_M6
P. crustosum
(b)
P. paneum LGAM SOW_M8
P. paneum CBS 101032
P. paneum LGAM SOW_M15
P. paneum
sect. Roquefortorum
81%
P. psychrosexuale CBS 128137
P. carneum CBS 112297
P. mediterraneum FMR 15188
P. roqueforti LGAM SOW_M5
P. roqueforti LGAM SOW_OM35
P. roqueforti CBS 221.30
P. roqueforti
genus Cladosporium
C. cladosporioides complex
C. austroafricanum CBS 140481
C. xylophilum CBS 125997
C. cladosporioides CBS 112388
C. cladosporioides LGAM SOW_M4
C. cladosporioides
96%
0.99
C. needhamense CPC 22353
C. phaenocomae CBS 128769
C. colombiae CBS 274.80B
C. herbarum complex
C. fildesense F09-T12-1
C. ramotenellum CBS 121628c
C. ramotenellum LGAM SOW_PT4
C. ramotenellum
(c)
C. tenellum CBS 121634
C. variabile CBS 121636
78%/0.95
C. prolongatum CGMCC3.18036
C. limoniforme LGAM SOW_OM52
C. limoniforme CBS 140484
C. limoniforme
genera Candida and Barnettozyma
B. xylosiphila NBRC 110202
B. hawaiiensis CBS 8760
94%
B. californica CBS 252
B. californica LGAM SOW_Y4
B. californica
C. oleophila CBS 2219
O. haglerorum VKPM Y-2583
C. chumphonensis CBS 12096
(d)
88%
0.98
C. boidinii CBS 2428
C. boidinii LGAM SOW_Y10
C. boidinii LGAM SOW_Y6
84%
C. boidinii LGAM SOW_Y7
C. boidinii CBS 8106
C. boidinii LGAM SOW_Y5
C. boidinii

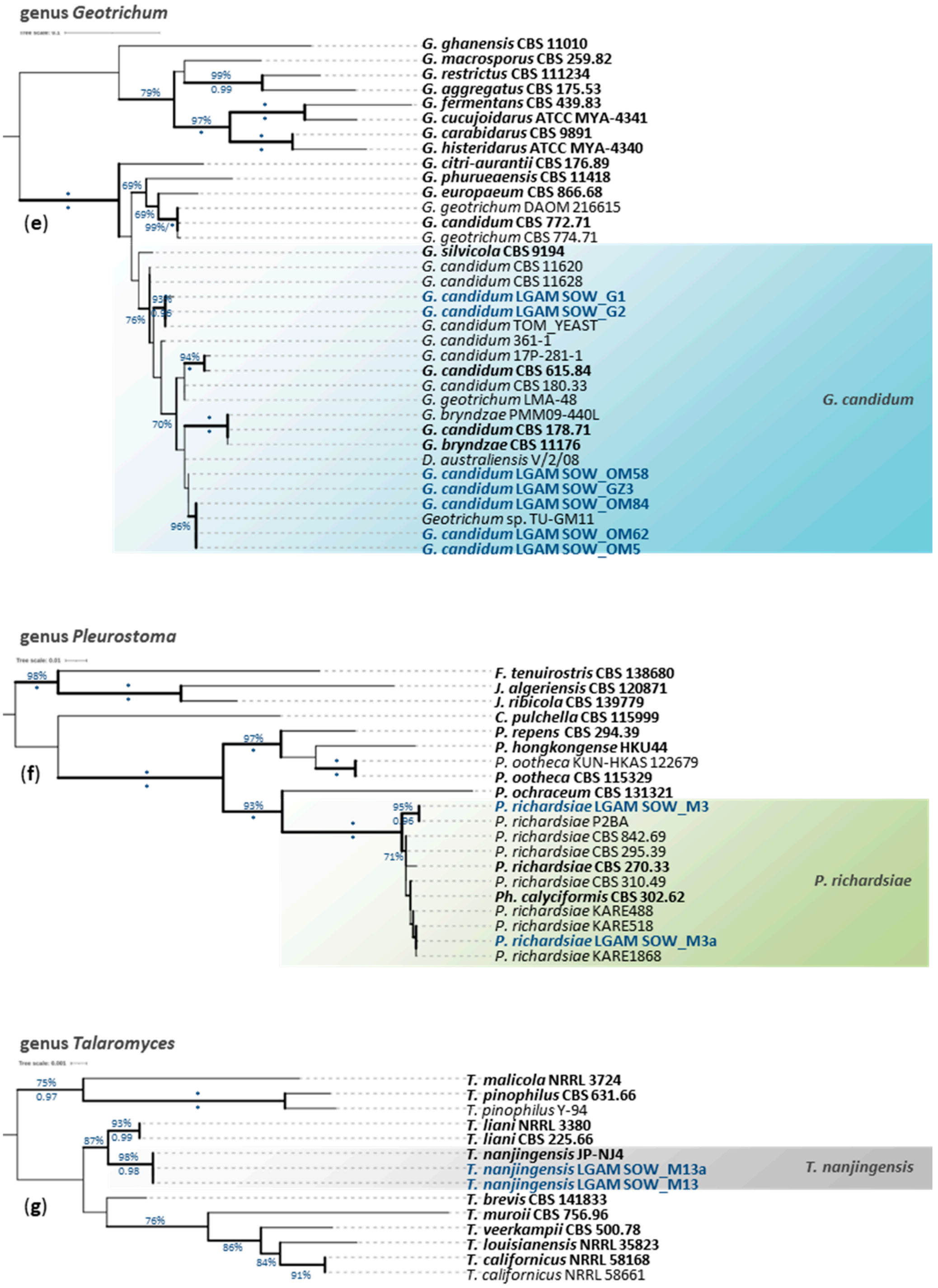

Figure S2. Phylogenetic trees for fungal genera/species corresponding to strains (representing MOTUs) of Ascomycota recovered from TPOMW. Molecular markers and the respective datasets used appear in Table 1 and in Supplementary Material, Table S3. Trees are presented in alphabetical order on the basis of the family name (Table 1): (**a**) *Aspergillus* (ITS and *tub*2; dataset DS_{ASP}); (**b**) *Penicillium* (ITS and *tub*2; DS_{PEN}); (**c**) *Cladosporium* (ITS and *act*; DS_{CLA}); (**d**) *Candida boidinii* and *Barnettozyma californica* (ITS and *tef*1-α; $DS_{CA\text{-}BA}$); (**e**) *Geotrichum* (ITS; DS_{GEO}); (**f**) *Pleurostoma* (ITS, 28S, *tub*2, *tef*1-α and *rpb*2; DS_{PLE}); (**g**) *Talaromyces* (ITS, *tub*2; DS_{TAL}). Species identified are presented in colored boxes. Type strains are shown in bold black, while strains obtained in this study are marked in bold blue. Intrageneric sections are indicated along major branches. Branch support values are shown where MLBS ≥ 65% and BPP ≥ 0.95; asterisks (*) denote MLBS = 100% and BPP = 1.00.
